# IMC29 Plays an Important Role in *Toxoplasma* Endodyogeny and Reveals New Components of the Daughter-Enriched IMC Proteome

**DOI:** 10.1128/mbio.03042-22

**Published:** 2023-01-09

**Authors:** Peter S. Back, Andy S. Moon, Rebecca R. Pasquarelli, Hannah N. Bell, Juan A. Torres, Allan L. Chen, Jihui Sha, Ajay A. Vashisht, James A. Wohlschlegel, Peter J. Bradley

**Affiliations:** a Molecular Biology Institute, University of California, Los Angeles, California, USA; b Department of Molecular Microbiology and Immunology, University of California, Los Angeles, California, USA; c Department of Biological Chemistry, David Geffen School of Medicine, University of California, Los Angeles, California, USA; University of Pittsburgh

**Keywords:** *Toxoplasma gondii*, inner membrane complex, coiled-coil domains, replication, parasitology, BioID

## Abstract

The *Toxoplasma* inner membrane complex (IMC) is a unique organelle that plays critical roles in parasite motility, invasion, egress, and replication. The IMC is delineated into the apical, body, and basal regions, defined by proteins that localize to these distinct subcompartments. The IMC can be further segregated by proteins that localize specifically to the maternal IMC, the daughter bud IMC, or both. While the function of the maternal IMC has been better characterized, the precise roles of most daughter IMC components remain poorly understood. Here, we demonstrate that the daughter protein IMC29 plays an important role in parasite replication. We show that Δ*imc29* parasites exhibit severe replication defects, resulting in substantial growth defects and loss of virulence. Deletion analyses revealed that IMC29 localization is largely dependent on the N-terminal half of the protein containing four predicted coiled-coil domains while IMC29 function requires a short C-terminal helical region. Using proximity labeling, we identify eight novel IMC proteins enriched in daughter buds, significantly expanding the daughter IMC proteome. We additionally report four novel proteins with unique localizations to the interface between two parasites or to the outer face of the IMC, exposing new subregions of the organelle. Together, this work establishes IMC29 as an important early daughter bud component of replication and uncovers an array of new IMC proteins that provides important insights into this organelle.

## INTRODUCTION

Toxoplasma gondii is a member of the phylum *Apicomplexa*, which consists of obligate intracellular parasites of medical and veterinary importance (1). These include Plasmodium falciparum (malaria), *Cryptosporidium* spp. (diarrheal disease), as well as the animal pathogens *Eimeria* spp., *Theileria* spp., *Babesia* spp., and Neospora caninum (2–6). Toxoplasma gondii infects nearly one-third of the world’s human population (7). While most infections are asymptomatic, the parasite can cause severe disease in immunocompromised people and congenitally infected neonates. To infect their host cells and cause disease, apicomplexan parasites utilize a set of specialized organelles. One of these is the inner membrane complex (IMC), which plays critical roles in many aspects of the parasite’s life cycle, including motility, invasion, replication, and egress (8–10).

In *Toxoplasma*, the IMC extends along the length of the parasite periphery and is composed of two layers: a series of membrane plates and a supporting network of cytoskeletal proteins (11, 12). These layers are further segregated into three compartments: the cone-shaped apical cap, a central body portion, and the posterior basal complex. Consistent with these distinct subdomains, IMC proteins often localize exclusively to one of these subcompartments and associate with the membrane plates, the cytoskeletal network, or span both (13, 14). Moreover, previous studies have identified proteins that localize exclusively to the maternal IMC, the daughter IMC, or both, further increasing the number of subcompartments (14, 15). Such distinct partitioning suggests that these IMC subdomains and their corresponding proteins provide discrete functions for the parasite.

Thus far, the IMC has three known functions. First, the IMC is an anchor for the glideosome, an actomyosin motor complex that powers gliding motility, which is necessary for both parasite invasion and egress (16, 17). Second, the apical cap of the IMC plays critical roles in stabilizing the apical complex to govern motility, invasion, and egress (9, 10, 18). Finally, the IMC serves as a scaffold for daughter cell assembly through a unique replication process called endodyogeny (8). In this mechanism of replication, budding daughter cells develop within the maternal cytoplasm, eventually replacing the degrading maternal IMC and adopting the expanding maternal plasma membrane to produce two fully formed parasites. While some recycling of the maternal IMC occurs, most daughter IMC components are synthesized and added onto forming daughter buds in a sequential, “just-in-time” manner (19, 20).

Consistent with this regulated approach, IMC proteins are not only categorized by their subcompartmental localization, but also by their timing of expression. To date, the earliest daughter IMC proteins include IMC32, FBXO1, and IMC15, which are expressed almost exclusively in the daughter buds (15, 21, 22). Other early daughter IMC proteins such as the AC9:AC10:ERK7 complex and ISP1 are present in the apical cap of both mother and daughter parasites (9, 10, 18, 23). In addition, the cytoskeletal proteins IMC1, IMC3, IMC4, IMC6, and IMC10 are expressed later in the replication process while IMC7, IMC12, and IMC14 are expressed exclusively in the maternal IMC (15, 24). Of the daughter-exclusive IMC proteins, FBXO1 was shown to be involved in IMC organization and maturation while IMC32 was demonstrated to be essential for assembling the daughter IMC during replication (21, 22). However, given the complex nature of endodyogeny, other uncharacterized daughter bud proteins are likely involved in facilitating the proper formation of the IMC.

In this study, we assessed the function of IMC29, one of the first IMC proteins identified to be highly enriched in forming daughter buds (14). We established that IMC29 is expressed at a similar time to other early daughter IMC proteins, contributing to the growing number of IMC proteins that likely initiate replication. We showed that disrupting IMC29 causes replication defects, resulting in severe growth defects and decreased virulence *in vivo*. Using deletion analyses, we showed that the predicted N-terminal palmitoylation site is dispensable and attribute the localization of IMC29 primarily to the N-terminal half of the protein containing four predicted coiled-coil domains. In addition, we demonstrated that a short helical region in the C terminus of the protein is critical for IMC29 function. We then utilized proximity labeling to identify 21 novel IMC proteins, eight of which localize to the daughter IMC. We additionally discovered a unique IMC-associated region by localizing four proteins found primarily in the interface between two intracellular parasites or on the outer face of the IMC. Together, we demonstrate the importance of IMC29 for parasite replication and reveal new cohorts of IMC proteins that further our understanding of this crucial organelle.

## RESULTS

### IMC29 is expressed early during endodyogeny.

**FIG 1:**
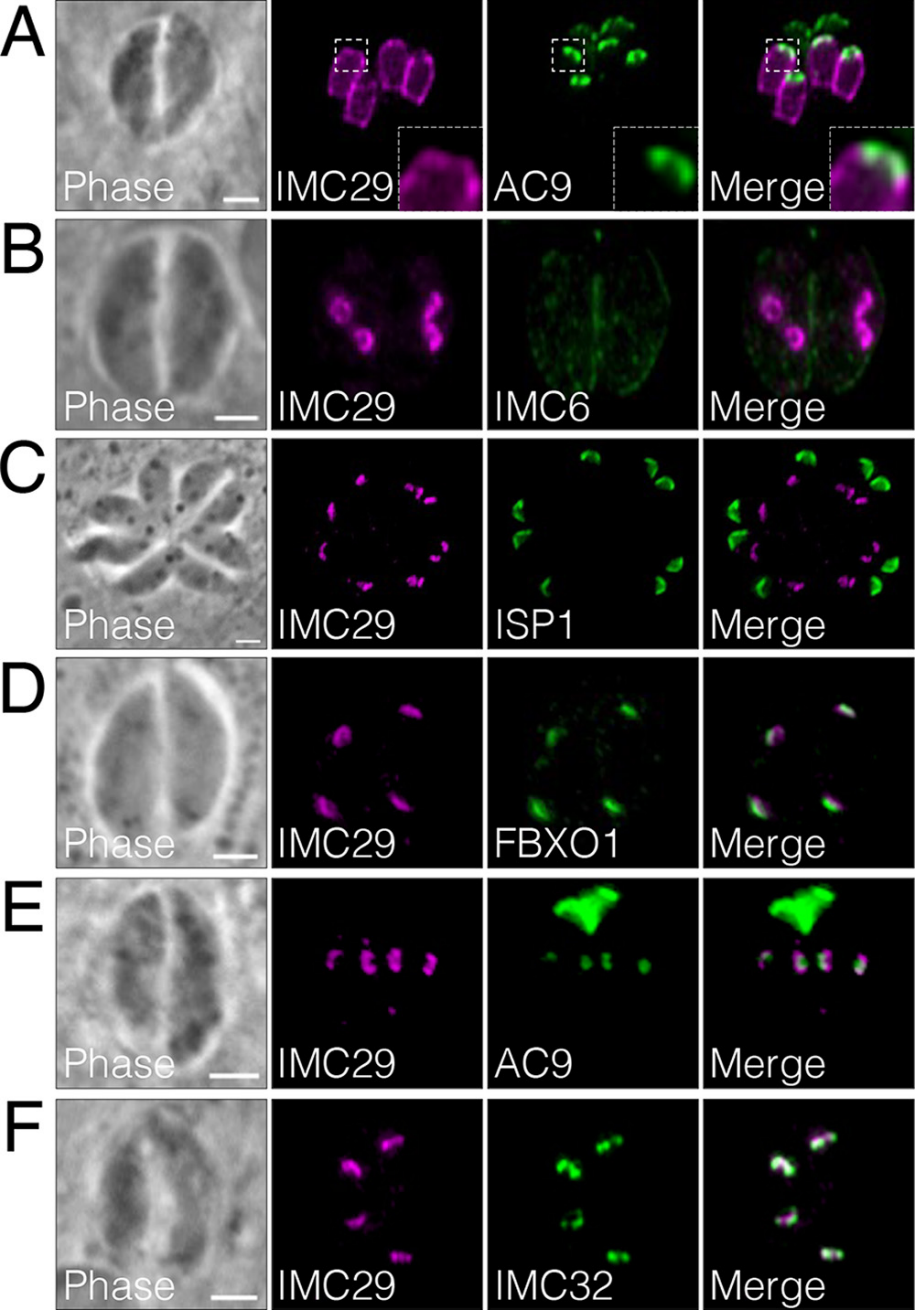
IMC29 is one of the earliest proteins expressed during endodyogeny. (A) Immunofluorescence assay (IFA) showing large daughter buds costained with IMC29 and the apical cap protein AC9. Insets were magnified 6.5-fold to highlight the lack of colocalization. Magenta, rabbit anti-HA; green, mouse anti-V5. (B) IMC29 costained with the daughter-enriched alveolin IMC6 shows that IMC29 is not present in the maternal IMC and appears before IMC6. Magenta, mouse anti-HA; green, rabbit anti-IMC6. (C) IMC29 appears in daughter buds before ISP1, which localizes to the apical cap of both maternal and daughter parasites. Magenta, rabbit anti-HA; green, mouse anti-ISP1. (D) IFAs show that IMC29 appears at approximately the same time as FBXO1. Magenta, mouse anti-Ty, green, rabbit anti-HA. (E) IMC29 appears around the same time as the cytoskeletal apical cap protein AC9. Magenta, rabbit anti-HA; green, mouse anti-V5. (F) IMC29 is expressed at a similar time to the essential daughter-enriched protein IMC32. Magenta, rabbit anti-HA; green, mouse anti-Ty. All scale bars are 2 μm.

IMC29 (TgGT1_243200) was initially identified in our ISC4-BioID studies and found to be enriched in the IMC of daughter buds (14). To better characterize the localization of IMC29, we endogenously tagged IMC29 with a 3xHA epitope tag (IMC29^3xHA^). We used this strain to first compare the localization of IMC29 to that of AC9, which showed that IMC29 is largely absent from the apical cap and restricted to the body portion of the daughter IMC (Fig. 1A). To determine its timing of expression more precisely, we compared IMC29 with a panel of known IMC proteins expressed early during endodyogeny. We found that IMC29 is expressed earlier than IMC6 and ISP1, which are present in both maternal and daughter parasites (Fig. 1B and C). We additionally observed that IMC29 is expressed as early as FBXO1 and AC9, apical cap proteins that are considered two of the earliest components of daughter cells (Fig. 1D and E) (9, 10, 21). Finally, we found that IMC29 is expressed at a similar time and colocalized well with IMC32, an IMC body protein that is also among the earliest expressed and is essential for daughter bud formation (Fig. 1F) (22). Together, this demonstrates that IMC29 is expressed at the initial stages of replication, suggesting a role in constructing the early scaffold of the daughter IMC.

### IMC29 is dispensable for invasion and egress but important for proper replication.

**FIG 2:**
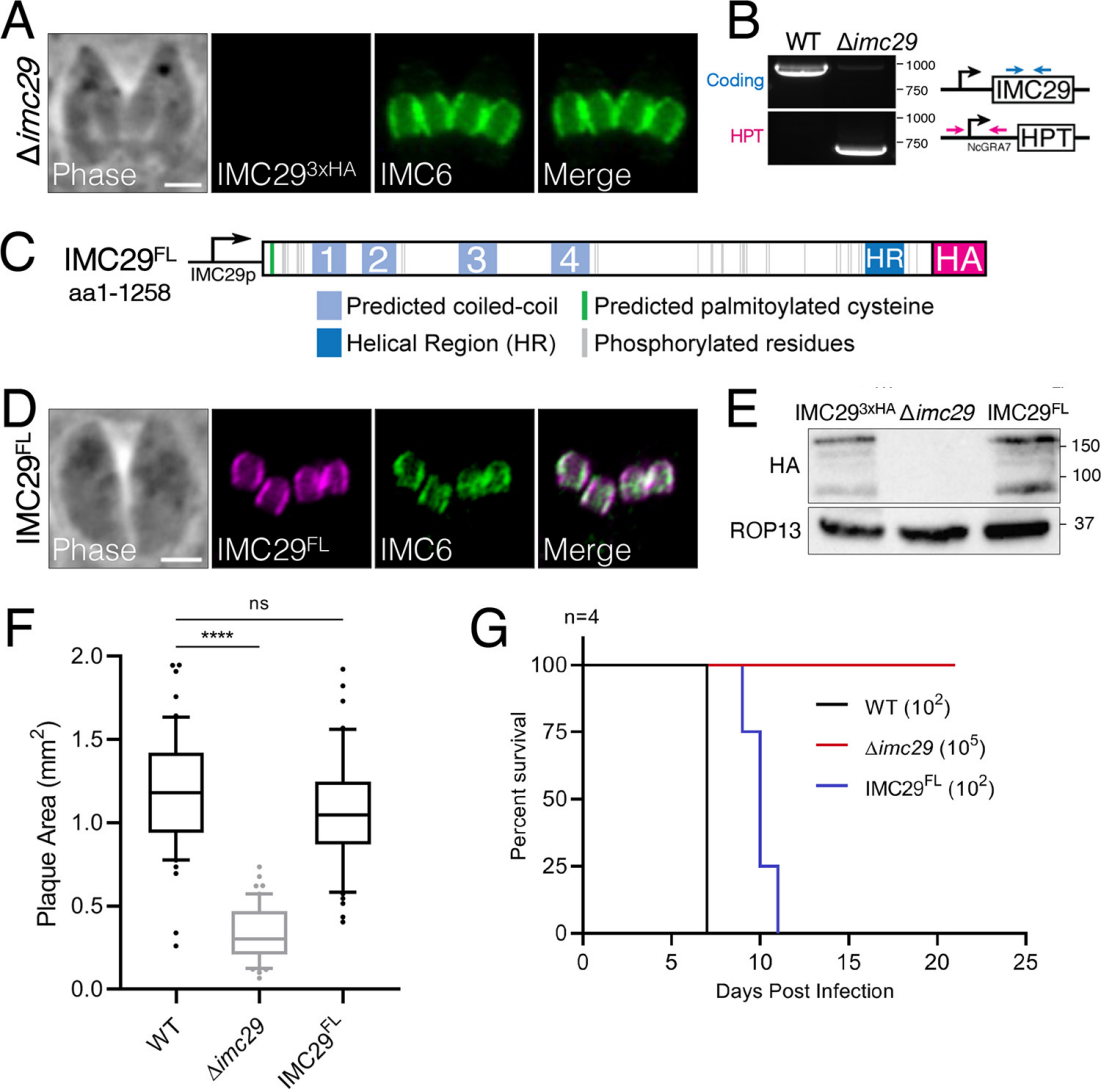
IMC29 is important for parasite fitness *in vitro* and *in vivo*. (A) IFA of Δ*imc29* parasites shows the absence of endogenous IMC29. Magenta, mouse anti-HA; green, rabbit anti-IMC6. (B) PCR verification for genomic DNA of WT (RHΔ*hxgprt*) and Δ*imc29* parasites. Diagram illustrates primers used to amplify the IMC29 coding sequence (blue arrows) and the site of recombination for the knockout locus (magenta arrows). (C) Diagram of full-length IMC29 (1,258 amino acids) driven by the endogenous promoter (denoted as IMC29^FL^), highlighting the predicted N-terminal palmitoylated cysteine at position 19, 32 phosphorylation sites, 4 predicted coiled-coil (CC) domains, a helical region (HR), and a 3xHA C-terminal epitope tag. (D) IFA of IMC29^FL^-complemented parasites shows restoration of daughter bud IMC localization. Magenta, mouse anti-HA; green, rabbit anti-IMC6. (E) Western blot of whole-cell lysates of IMC29^3xHA^, Δ*imc29*, and IMC29^FL^ parasites showing the absence of endogenous IMC29 and restored levels of complemented IMC29^FL^. IMC29 was detected with mouse anti-HA; ROP13 was used as a loading control and detected with mouse anti-ROP13. (F) Quantification of plaque assays depicts the severe growth defect of Δ*imc29* parasites, which is completely rescued by the full-length complementation construct. Significance was determined using multiple two-tailed *t* tests. ****, *P* < 0.0001; ns, not significant (*P* = 0.1957). (G) Mouse virulence assay illustrates the sharp decrease in virulence of Δ*imc29* parasites (red) compared to wild-type parasites (black) and IMC29^FL^ parasites (blue). All scale bars are 2 μm.

IMC29 was assigned a severe fitness score of −3.95 in the T. gondii genome-wide CRISPR screen (GWCS), indicating that it is important or essential for the parasite (25). To assess the function of IMC29, we disrupted its gene from the IMC29^3xHA^ strain to obtain Δ*imc29* parasites. The knockout was verified by the lack of staining for the endogenously tagged protein and by PCR (Fig. 2A and B). We then generated a full-length, 3xHA-tagged IMC29 complementation construct driven by its endogenous promoter and targeted to the UPRT locus (IMC29^FL^) (Fig. 2C). We expressed this construct in Δ*imc29* parasites and performed immunofluorescence assay (IFA) and Western blot analyses to show that IMC29^FL^ restores daughter IMC localization and expression similar to wild-type levels (Fig. 2D and E). We then evaluated the overall lytic ability of Δ*imc29* parasites by plaque assay, which demonstrated a 71.2% decrease in plaque size compared to wild-type parasites (Fig. 2F). In addition, we examined virulence by injecting mice intraperitoneally with either 10^2^ wild-type, 10^5^ Δ*imc29*, or 10^2^ IMC29^FL^ parasites (Fig. 2G). While the mice inoculated with either the wild-type or IMC29^FL^ parasites died between 7 and 11 days postinfection, those inoculated with Δ*imc29* parasites survived the infection, showing a dramatic 1,000-fold decrease in virulence. Together, these data demonstrate that IMC29 is important for parasite fitness *in vitro* and *in vivo*.

**FIG 3:**
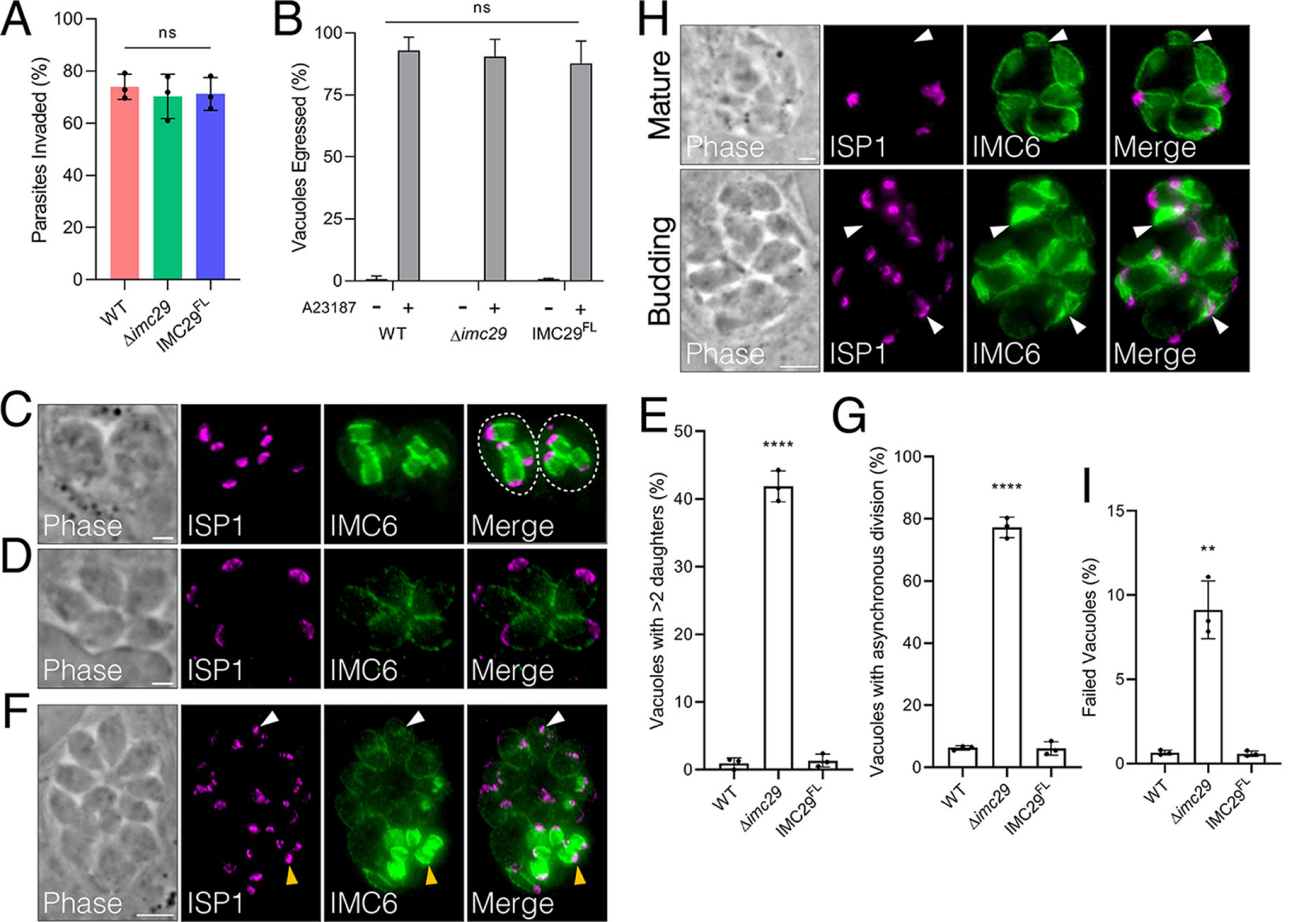
Disrupting IMC29 causes severe replication defects. (A) Red-green invasion assays showing no invasion defect for Δ*imc29* parasites compared to wild-type or IMC29^FL^ parasites. Triplicate experiments were performed with >400 individual parasites counted per replicate, and significance was calculated using multiple two-tailed *t* tests. (B) Calcium-induced egress assays displaying no egress defect upon knockout of IMC29. A23187: calcium ionophore. Triplicate experiments were performed by counting >200 total vacuoles counted across at least 10 different fields per replicate. Significance was determined using two-way ANOVA and Tukey’s multiple-comparison test. (C) Representative IFA shows an abnormal number of daughter buds (three or four) within each maternal cell. Magenta, mouse anti-ISP1; green, rabbit anti-IMC6. (D) Representative IFA showing an atypical number (five) of mature parasites. Magenta, mouse anti-ISP1; green, rabbit anti-IMC6. (E) Quantification of vacuoles containing more than two daughter buds per dividing parasite at 24 h postinfection. Triplicate experiments were performed by counting >150 total vacuoles counted across at least 15 different fields per replicate. Significance was determined with multiple two-tailed *t* tests. ****, *P* < 0.0001. (F) Representative IFA depicts a large vacuole undergoing division asynchronously with some daughter buds at much later stages (yellow arrowheads) and some just beginning to divide (white arrowheads). Magenta, mouse anti-ISP1; green, rabbit anti-IMC6. Scale bar: 5 μm. (G) Quantification of vacuoles exhibiting asynchronous division at 32 h postinfection. Triplicate experiments were performed with >200 total vacuoles counted across at least 15 different fields per replicate. Significance was determined with multiple two-tailed *t* tests. ****, *P* < 0.0001. (H) Representative IFA shows examples of mature and budding vacuoles with gross morphological defects. Top: arrowheads highlight parasites without an apical cap as assessed by ISP1. (scale bar: 2 μm). Bottom: arrowheads indicate collapsed daughter buds (scale bar: 5 μm). Magenta, mouse anti-ISP1; green, rabbit anti-IMC6. (I) Quantification of vacuoles with gross morphological defects at 32 h postinfection. Triplicate experiments were performed by counting >400 total vacuoles across at least 15 different fields per replicate. Significance was determined with multiple two-tailed *t* tests. **, *P* = 0.001. All scale bars are 2 μm unless otherwise indicated.

The expression timing and localization of IMC29 suggest that it is likely involved in the early stages of replication. Consistent with this hypothesis, we found that Δ*imc29* parasites have no significant defects in invasion or egress (Fig. 3A and B). To assess the fidelity of replication, we stained intracellular parasites with the IMC markers ISP1 and IMC6 and noticed several abnormal phenotypes. First, we frequently observed Δ*imc29* vacuoles containing more than 2 daughter buds (often 3 or 4) per maternal cell (Fig. 3C), resulting in an atypical number of mature parasites (Fig. 3D). Quantifying vacuoles with >2 daughter buds demonstrated that 41.9 ± 2.3% of Δ*imc29* vacuoles exhibited this phenotype compared to the low level of error in wild-type vacuoles (0.94 ± 0.82%), suggesting a dysregulation in controlling the number of daughter buds (Fig. 3E). In agreement with this, a high frequency of Δ*imc29* vacuoles (77.3 ± 3.3%) displayed uncoordinated division, resulting in daughter buds at drastically different stages of maturation within one vacuole (Fig. 3F and G). We also observed Δ*imc29* parasites with gross morphological defects such as mature parasites with missing apical caps or budding parasites with collapsed daughter buds, suggesting that these parasites may have failed to divide (Fig. 3H). We quantified this by counting any Δ*imc29* vacuole exhibiting such morphological defects, which showed a modest but reproducible increase of 9.1 ± 1.7% for this phenotype compared to 0.66 ± 0.14% for wild-type parasites (Fig. 3I). Importantly, all replication defects were completely rescued in the IMC29^FL^ parasites, indicating that IMC29 is directly responsible for these defects.

We then stained Δ*imc29* parasites for various organelles, including the apicoplast, plant-like vacuole (PLV/VAC), micronemes, and rhoptries, which all appeared unperturbed (see Fig. S1 in the supplemental material). Surprisingly, staining for the mitochondria showed collapsed morphology rather than the typical lasso shape (Fig. S2C). However, we found that this mitochondrial defect was also present in IMC29^3xHA^ parasites, indicating that the tagging process introduced an unintended effect on the mitochondria that carried over to the Δ*imc29* and subsequently the IMC29^FL^ parasites (Fig. S2A to D). Importantly, however, we had shown that IMC29^FL^ parasites fully rescued the defects incurred by disrupting IMC29, including plaque defects, virulence, and replication errors. Thus, despite the inadvertent effects on the mitochondria, these replication phenotypes are directly linked to IMC29 and the off-target effects on the mitochondria are irrelevant to this study.

10.1128/mbio.03042-22.1 FIG S1 The apicoplast, PLV/VAC, micronemes, and rhoptries are unaffected in Δ*imc29* parasites. All IFAs show normal morphology of the indicated organelles. (A) The apicoplast was detected using mouse anti-ATrx1 (magenta). Scale bar: 5 μm. (B) The PLV/VAC was detected with guinea pig anti-NHE3 (magenta). (C) Micronemes were detected with mouse anti-MIC2 (magenta). (D) Rhoptries were detected with mouse anti-ROP7 (magenta). All IFAs were costained with rabbit anti-IMC6 (green). Scale bars for (B–D) are 2 μm. Download FIG S1, TIF file, 2.7 MB. Copyright © 2023 Back et al. 2023 Back et al. https://creativecommons.org/licenses/by/4.0/ This content is distributed under the terms of the Creative Commons Attribution 4.0 International license.

10.1128/mbio.03042-22.2 FIG S2 Epitope tagging of endogenous IMC29 caused an inadvertent effect on mitochondrial morphology. (A–D) IFAs showing intact mitochondrial morphology of wild-type parasites (A) compared to the endogenously tagged (B), knockout (C), and complemented parasites (D). Magenta, mouse anti-F_1_β; green, rabbit anti-IMC6. (E) IFA of regenerated IMC29^3xHA^ parasites showing typical daughter IMC staining. Magenta, mouse anti-HA; green, rabbit anti-IMC6. (F) IFA of knockout parasites regenerated from the new IMC29^3xHA^ strain showing absence of the endogenous protein. Magenta, mouse anti-HA; green, rabbit anti-IMC6. (G) PCR verification for genomic DNA of WT (RHΔ*hxgprt*) and new Δ*imc29*^regenerated^ parasites. Diagram shows primers used to amplify the IMC29 coding sequence (blue arrows) and the site of recombination for the knockout locus (magenta arrows). (H) IFA of regenerated tagged parasites showing typical lasso-shaped mitochondria. Magenta, mouse anti-F_1_β; green, rabbit anti-IMC6. (I) IFA of new Δ*imc29*^regenerated^ parasites showing normal lasso-shaped mitochondria. Magenta, mouse anti-F_1_β; green, rabbit anti-IMC6. (J) Plaque assays for the new IMC29^3xHAregenerated^ and Δ*imc29*^regenerated^ parasites compared to WT, Δ*imc29*^PC^, and complemented strains. Significance was determined using multiple two-tailed *t*-tests. ****, *P* < 0.0001. All scale bars are 2 μm. Download FIG S2, JPG file, 0.7 MB. Copyright © 2023 Back et al. 2023 Back et al. https://creativecommons.org/licenses/by/4.0/ This content is distributed under the terms of the Creative Commons Attribution 4.0 International license.

We supported this claim by regenerating IMC29^3xHA^ parasites and using this new strain to knock out *IMC29* with a different guide RNA (gRNA) and a different homology-directed repair template (Δ*imc29*^regenerated^) (Fig. S2E to G). These new strains did not exhibit any mitochondrial defects and largely recapitulated the growth defects observed in the original lines (Fig. S2H to J). This underscores the importance of IMC29 for parasite fitness and demonstrates that the fitness defect is not linked to the mitochondria. Taken together, these findings indicate that IMC29 serves as a key component of the daughter cell scaffold during the earliest stages of endodyogeny.

### Δ*imc29* parasites partially compensate after extended passaging.

**FIG 4:**
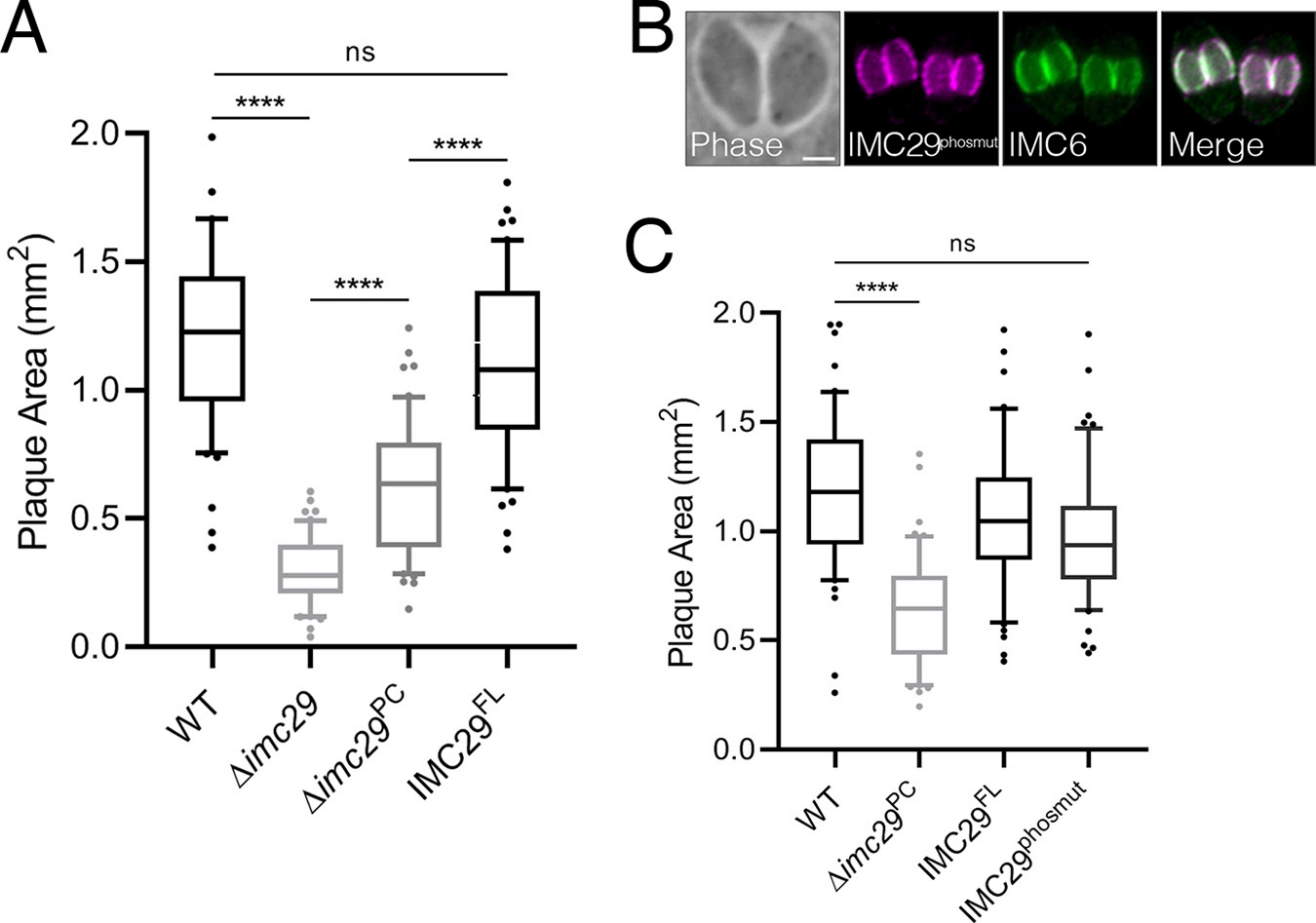
Δ*imc29* parasites partially compensate and IMC29 does not depend on phosphorylation for localization or function. (A) Plaque assay quantification shows that Δ*imc29*^PC^ (partially compensated) parasites exhibit a less substantial plaque defect compared to Δ*imc29* parasites. Significance was calculated using multiple two-tailed *t* tests. ****, *P* < 0.0001; ns, not significant (*P* = 0.0968). (B) IFA shows proper localization of IMC29^phosmut^ to the daughter IMC periphery. Magenta, mouse anti-HA; green, rabbit anti-IMC6. Scale bar: 2 μm. (C) Plaque assay shows that IMC29^phosmut^ rescues the plaque defect. Significance was determined with multiple two-tailed *t* tests. ****, *P* < 0.0001; ns, not significant (*P* = 0.1072).

We observed an interesting phenomenon after passaging the original Δ*imc29* parasites for several months in culture, during which they partially recovered their fitness defect and produced a less severe plaque defect (denoted as Δ*imc29* partially compensated, Δ*imc29*^PC^). Notably, the Δ*imc29*^PC^ parasites did not compensate further even after an additional year in culture and still exhibited a significant reduction in plaque size compared to wild-type parasites (Fig. 4A). To determine if compensation could be avoided, we conditionally depleted IMC29 using an auxin-inducible degron (IMC29^AID^). Upon addition of auxin, IMC29 was knocked down to undetectable levels by IFA and Western blot (Fig. S3A and B). However, plaque assays demonstrated that depleting IMC29 only results in a modest 33.2% decrease in plaque size (Fig. S3C). This indicates that AID-mediated depletion does not capture the full extent of defects incurred by a direct knockout and suggests that the knockdown is incomplete, allowing residual amounts of IMC29 to perform its functions. Thus, rather than using the IMC29^AID^ or Δ*imc29*^regenerated^ parasites which may still undergo compensation, we reasoned that the original Δ*imc29*^PC^ parasites provide the best background to functionally evaluate IMC29 domains and features as it still produced a stable and significant plaque defect.

10.1128/mbio.03042-22.3 FIG S3 AID-mediated depletion is not sufficient to reproduce the severe defects of Δ*imc29*. (A) IFAs of IMC29^AID^ showing auxin-induced depletion of IMC29. Magenta, mouse anti-HA; green, rabbit anti-IMC6. (B) Western blot of whole-cell lysates of IMC29^AID^–/+ IAA harvested after 30 h. IMC29^AID^ was detected with mouse anti-HA; RON5C was used as a loading control and detected with rabbit anti-RON5C. (C) Plaque assay to compare growth defects of IMC29^AID^ parasites with and without IAA. Significance was determined using a two-tailed *t*-test. ****, *P* < 0.0001. (D) IFAs of IMC29^AID^ showing intact mitochondrial morphology regardless of IMC29 depletion. Magenta, mouse anti-F_1_β; green, rabbit anti-IMC6. All scale bars are 2 μm. Download FIG S3, TIF file, 2.7 MB. Copyright © 2023 Back et al. 2023 Back et al. https://creativecommons.org/licenses/by/4.0/ This content is distributed under the terms of the Creative Commons Attribution 4.0 International license.

### IMC29 localization is largely dependent on the N-terminal region containing four predicted coiled-coil domains.

Like many other IMC proteins, IMC29 is relatively large (1,258 amino acids) and lacks homology to known proteins. However, it contains several features that may be important for localization and function, including 32 phosphorylated serine/threonine residues identified by phosphoproteomics and a predicted palmitoylated residue at Cys19 (26, 27). It also contains four predicted coiled-coil (CC) domains in the N-terminal half of the protein and a weakly predicted CC domain near the C terminus of the protein that we designated as the Helical Region (HR) (Fig. S4) (28–31). To assess whether these IMC29 features are important for localization and function, we generated mutant and deletion complementation constructs to express them in the Δ*imc29*^PC^ parasites.

10.1128/mbio.03042-22.4 FIG S4 Prediction analyses for IMC29 coiled-coil domains. (A) The full IMC29 amino acid sequence was queried using the NPS@ server to generate the probability graph (29). The four N-terminal coiled-coil domains were sectioned based on a probability threshold of 0.5 on at least one of the three windows. Based on this, CC1 contains residues 107 to 145, CC2 contains residues 168 to 198, CC3 contains residues 346 to 376, and CC4 contains residues 451 to 492. The C-terminal helical region (HR) contains residues 1118 to 1147 and was named due to its high level of conservation with orthologues from other coccidian parasites despite its weaker scores for coiled-coil probability. (B) The N-terminal residues 1 to 499 were queried using DeepCoil (sequence length is limited to 500 by the software) (30, 31). Probability graph largely corroborates the predictions made using NPS@, denoted by the CC1-4 designations. (C) The C-terminal residues 760 to 1258 were queried using DeepCoil. Probability graph shows a peak at residues 1118 to 1147, similar to the NPS@ server. Download FIG S4, JPG file, 0.6 MB. Copyright © 2023 Back et al. 2023 Back et al. https://creativecommons.org/licenses/by/4.0/ This content is distributed under the terms of the Creative Commons Attribution 4.0 International license.

**FIG 5:**
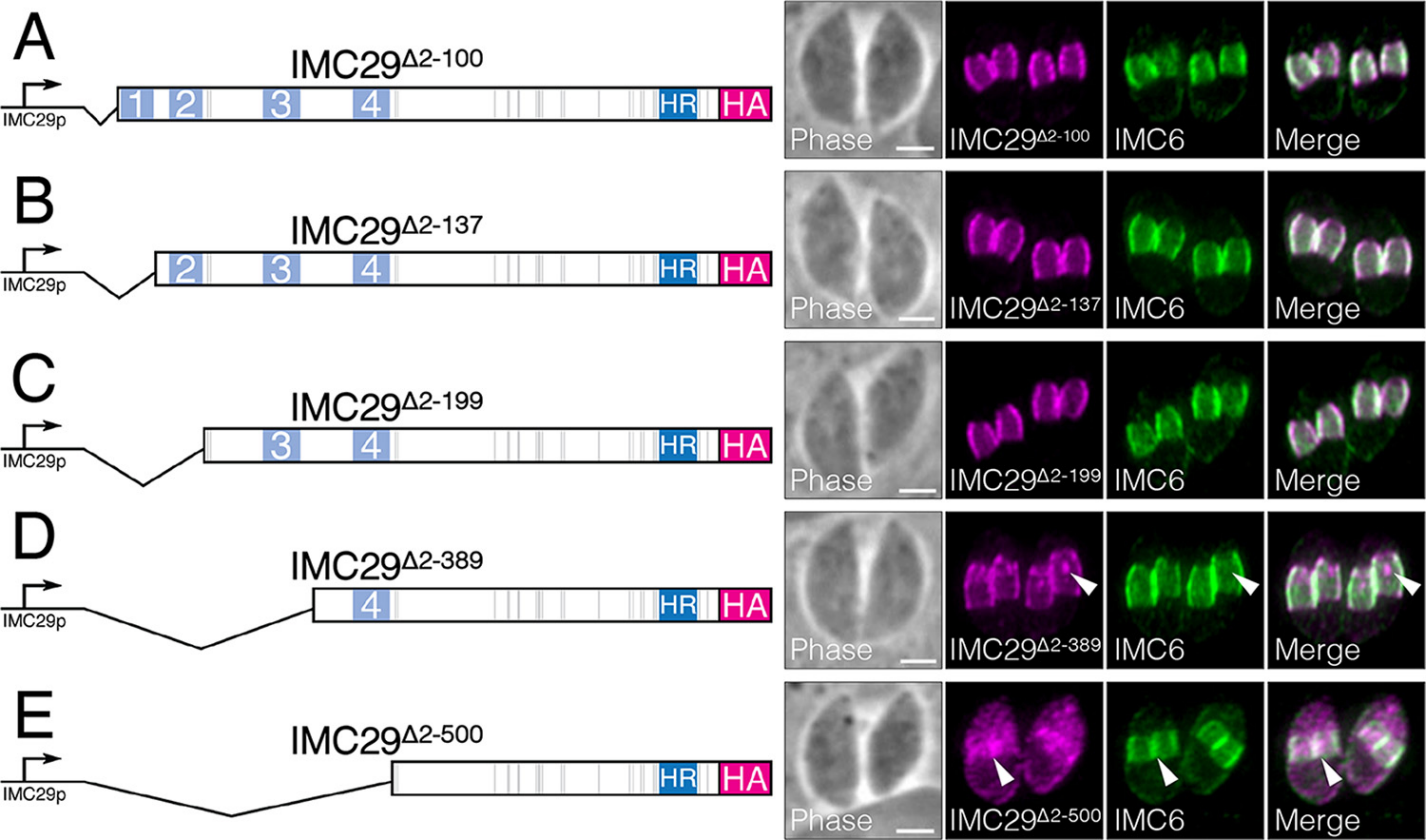
IMC29 localization is largely dependent on the N-terminal half of the protein. Diagrams and IFAs of each N-terminal deletion complementation construct. Each construct is driven by the endogenous promoter, C-terminally tagged with 3xHA, and expressed in Δ*imc29*^PC^ parasites. For all IFAs: magenta, mouse anti-HA; green, rabbit anti-IMC6. (A) IFA of IMC29^Δ2-100^, which includes the predicted palmitoylated cysteine, shows that the daughter IMC localization is unaffected. (B) IFA of IMC29^Δ2-137^ which includes the first predicted CC domain or (C) IFA of IMC29^Δ2-199^ which includes two predicted CC domains both show that the daughter IMC localization is unaffected. (D) IFA of IMC29^Δ2-389^, including three predicted CC domains, shows that peripheral staining of daughter IMC is mostly retained but some mislocalized material often appears as a single, concentrated spot in the cytoplasm of daughter buds (arrowheads). (E) IFA of IMC29^Δ2-500^, including all four predicted N-terminal CC domains, shows severe mislocalization to the cytoplasm of the dividing maternal parasite, though some staining at the daughter periphery is still enriched (arrowheads). All scale bars are 2 μm.

We first assessed the role of phosphorylation by mutating all 32 known phosphorylation sites to alanine simultaneously (IMC29^phosmut^) and expressed this construct in Δ*imc29*^PC^ parasites. We found that IMC29^phosmut^ localized properly to the daughter IMC and rescued IMC29 function, indicating that phosphorylation at these residues is dispensable (Fig. 4B and C). We next focused on palmitoylation, as it has frequently been shown to mediate tethering of IMC proteins to the IMC membrane vesicles (22, 23, 32, 33). To determine whether the predicted palmitoylation at Cys19 is important for IMC29 localization, we deleted the N terminus of the protein up to the first CC domain (IMC29^Δ2-100^) and expressed it in Δ*imc29*^PC^ parasites. Surprisingly, we observed that IMC29^Δ2-100^ localization is unaffected (Fig. 5A). This indicates that, unlike other palmitoylated IMC proteins, IMC29 localization is not dependent on this predicted palmitoylation site, nor the entirety of the N-terminal portion of the protein.

To interrogate the rest of the N-terminal region encompassing four predicted CC domains, we generated a series of deletion constructs guided by coiled-coil prediction analyses, regions of conservation, and secondary structure predictions (28, 31, 34, 35). We started by creating a deletion that includes the first predicted CC domain (IMC29^Δ2-137^) as well as a deletion that includes the first two predicted CC domains (IMC29^Δ2-199^). Each deletion construct was expressed in Δ*imc29*^PC^ parasites and found to localize properly to the daughter IMC, indicating that these regions are dispensable for IMC29 localization (Fig. 5B and C). We then deleted the region containing the first three predicted CC domains (IMC29^Δ2-389^) and found that while the truncated protein mostly localized properly to the daughter IMC, we consistently observed a single concentrated spot of mislocalized IMC29^Δ2-389^ in the cytoplasm of the developing daughter buds (Fig. 5D). As this spot was reminiscent of the Golgi apparatus in dividing parasites, we colocalized IMC29^Δ2-389^ with the Golgi marker GRASP55-YFP and found that while they often appeared near each other, they did not colocalize (Fig. S5A) (36). Finally, we created a deletion that includes all four predicted N-terminal CC domains (IMC29^Δ2-500^). We found that IMC29^Δ2-500^ was substantially mislocalized to the cytoplasm of daughter buds, though some enrichment in the daughter IMC was retained (Fig. 5E). This demonstrates that while the regions containing the third and fourth CC domains are important for anchoring IMC29, the remaining C-terminal half of the protein likely also plays a role in localization.

10.1128/mbio.03042-22.5 FIG S5 IMC29^Δ2-389^ does not colocalize with the Golgi apparatus and the N-terminal half of IMC29 is dispensable for function. (A) IFAs of parasites transiently expressing GRASP55-YFP, showing that the concentrated spot of IMC29^Δ2-389^ does not colocalize with this Golgi marker. Arrowheads point to the mislocalized spots. Magenta, mouse anti-HA; green, GRASP55-YFP. (B) Plaque assays demonstrate that each N-terminal deletion construct rescues the plaque defect. Multiple two-tailed *t*-tests were performed to assess significance. ****, *P* < 0.0001. ns: *P* ≥ 0.05. Download FIG S5, TIF file, 1.3 MB. Copyright © 2023 Back et al. 2023 Back et al. https://creativecommons.org/licenses/by/4.0/ This content is distributed under the terms of the Creative Commons Attribution 4.0 International license.

We then performed plaque assays to determine the functional significance of each of these N-terminal deletions (Fig. S5B). Consistent with their localization, IMC29^Δ2-100^, IMC29^Δ2-137^, IMC29^Δ2-199^, and IMC29^Δ2-389^ rescued the plaque defect of the Δ*imc29*^PC^ strain, indicating that any subtle localization defects caused by these deletions do not significantly influence IMC29 function. Surprisingly, even IMC29^Δ2-500^ rescued the plaque defect, which indicates that the small amounts of IMC29 remaining in the daughter IMC is sufficient for IMC29 function, further supporting the hypothesis that the C terminus contains important regions for IMC29 localization and function.

### IMC29 function requires a short helical region in the C terminus.

**FIG 6:**
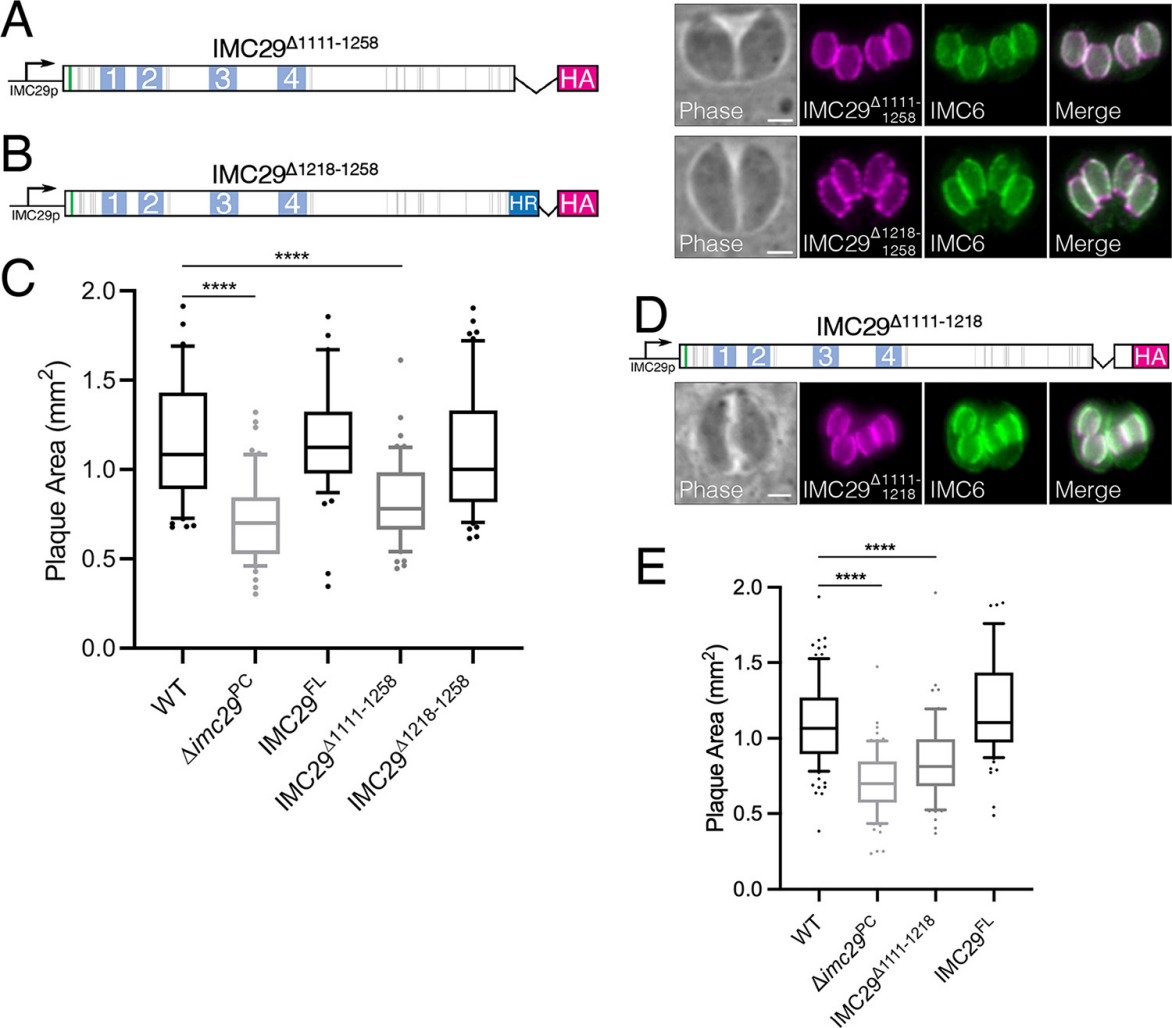
IMC29 function is dependent on a short C-terminal region. (A) Diagram and IFA of IMC29^Δ1111-1258^, which removes the C-terminal predicted helical region as well as the remaining C-terminal end, shows that daughter IMC localization remains intact. (B) Diagram and IFA of IMC29^Δ1218-1258^, which removes only the extreme C-terminal end, shows proper daughter IMC localization. (C) Plaque assays show that while IMC29^Δ1218-1258^ rescues the plaque defect, IMC29^Δ1111-1258^ does not. Significance was determined using multiple two-tailed *t* tests. ****, *P* < 0.0001. (D) Diagram and IFA of IMC29^Δ1111-1218^, which removes only the C-terminal predicted helical region, shows that daughter IMC localization is unaffected. (E) Plaque assay indicates that IMC29^Δ1111-1218^ cannot rescue IMC29 function. Significance was determined using multiple two-tailed *t* tests. ****, *P* < 0.0001. For all IFAs: magenta, mouse anti-HA; green, rabbit anti-IMC6. All scale bars are 2 μm.

To assess the role of the C terminus, we generated two deletion constructs and expressed them in Δ*imc29*^PC^ parasites. The first deletion removed the entire C-terminal portion of the protein, including the HR (IMC29^Δ1111-1258^), while the second construct removed only the region C-terminal to the HR (IMC29^Δ1218-1258^) (Fig. 6A and B). We found that both IMC29^Δ1111-1258^ and IMC29^Δ1218-1258^ localized properly to the daughter IMC, indicating that this region is not necessary for IMC29 localization. Intriguingly, IMC29^Δ1218-1258^ fully rescued the plaque defect but IMC29^Δ1111-1258^ did not, demonstrating that the HR is critical for IMC29 function (Fig. 6C). To assess the HR more directly, we generated an additional construct in which only the HR was deleted (IMC29^Δ1111-1218^). As expected, IMC29^Δ1111-1218^ localized properly to the daughter IMC (Fig. 6D). Importantly, plaque assays showed that IMC29^Δ1111-1218^ could not fully rescue the plaque defect, further supporting our hypothesis that the HR is largely responsible for IMC29 function (Fig. 6E). Together, our dissection of IMC29 domains indicates that localization and function are attributed to different regions of the protein — the N-terminal half contributes primarily to localize IMC29 to the daughter IMC and the C terminus is necessary for IMC29 to carry out its role in ensuring faithful parasite replication.

### IMC29 proximity labeling reveals new components of the daughter-enriched IMC.

The complex process of replication via endodyogeny likely requires the tightly regulated collaboration of many IMC proteins. While several daughter-specific proteins have been recently characterized, the proteome of the daughter buds is likely incomplete (14, 15, 21, 22, 37). Thus, we used IMC29 as the bait protein for *in vivo* biotinylation (BioID) to identify novel daughter-enriched IMC proteins. We endogenously tagged IMC29 with the promiscuous biotin ligase BirA* (Fig. S6A) and confirmed that the daughter IMC subcompartment was robustly biotinylated, demonstrating that the fusion protein was active (Fig. S6B and C).

10.1128/mbio.03042-22.6 FIG S6 IMC29^BirA^* robustly labels the daughter IMC subcompartment. (A) Diagram depicting the endogenous IMC29 fused to the biotin ligase BirA* and the 3xHA epitope tag. (B) IFA shows proper localization of the IMC29^BirA^* fusion protein to the daughter IMC without biotin. Endogenous streptavidin staining of the apicoplast is also shown. Magenta, mouse anti-HA; green, anti-streptavidin 488. (C) IFA shows biotin-dependent labeling of the daughter IMC subcompartment. Magenta, mouse anti-HA; green, anti-streptavidin 488. Scale bars are 2 μm. Download FIG S6, TIF file, 1.8 MB. Copyright © 2023 Back et al. 2023 Back et al. https://creativecommons.org/licenses/by/4.0/ This content is distributed under the terms of the Creative Commons Attribution 4.0 International license.

To identify targets labeled by IMC29^BirA^*, we conducted two separate BioID experiments. First, we performed the standard protocol by harvesting intracellular parasites and lysing them with radioimmunoprecipitation assay (RIPA) buffer to obtain whole-cell lysates. Second, we detergent extracted intracellular parasites to isolate a detergent-insoluble fraction, as we have shown that this modification enriches for IMC components (14). Samples prepared using either method were analyzed by mass spectrometry as previously described (14). Both data sets were ranked by spectral counts normalized by molecular weight (NSAF), and proteins common to both the control and IMC29-BirA* lysates were included if the ratio of IMC29 to control was greater than 2. The annotated list from the whole-cell lysates experiment is shown in Table S1 and the list from the detergent-insoluble experiment is shown in Table S2. As expected, the top hits from the detergent-insoluble fraction were significantly enriched for known IMC cytoskeletal proteins compared to the whole-cell lysate. Hypothetical proteins from both data sets were filtered based on IMC-typical cyclical expression patterns and localized by endogenous gene tagging. The summary of all localized proteins is shown in Table 1.

**TABLE 1:** Summary of verified proteins identified by IMC29-BioID

Localization	Gene ID	Protein name	GWCS score	Description (ToxoDB)
Daughter-enriched IMC	TgGT1_294610	IMC30	–0.98	Histone lysine methyltransferase, SET, putative
	TgGT1_255450	IMC31	–1.78	Hypothetical protein
	TgGT1_240630	IMC35	1.73	ULK kinase
	TgGT1_297870	IMC36	1.52	Hypothetical protein
	TgGT1_294860	BCC0	–4.1	Hypothetical protein
	TgGT1_311770	BCC3	–0.11	Hypothetical protein
Daughter apical cap	TgGT1_249440	AC12	–0.48	Hypothetical protein
	TgGT1_220900	AC13	–3.42	Hypothetical protein
IMC-associated proteins	TgGT1_310450	IAP2	–1.24	Putative myosin heavy chain
	TgGT1_238170	IAP3	–5.06	Hypothetical protein
	TgGT1_221630	IAP4	–1.97	Hypothetical protein
	TgGT1_224000	IAP5	–1.47	Hypothetical protein
Maternal IMC	TgGT1_235690	IMC37	1.12	Hypothetical protein
	TgGT1_293360	IMC38	0.11	Hypothetical protein
	TgGT1_255420	IMC39	1.74	Hypothetical protein
	TgGT1_269960	IMC40	–1.12	14-3-3 superfamily protein
	TgGT1_225560	IMC41	2.18	Hypothetical protein
	TgGT1_312100	IMC42	–0.42	Plasma membrane-type Ca(2+)-ATPase A1 PMCAA1
Basal complex	TgGT1_229260	BCC4	–3.52	Hypothetical protein
	TgGT1_202550	BCC6	–0.39	NLI interacting factor family phosphatase, CTDSPL3
	TgGT1_311230	BCC7	0.74	Hypothetical protein

10.1128/mbio.03042-22.8 TABLE S1 List of top hits identified by mass spectrometry of IMC29-BioID whole-cell lysates. Proteins were ranked by NSAF. Proteins present in the RH-Control were not excluded but only included in this list if the ratio of IMC29:RH > 2. Gene IDs and descriptions are from ToxoDB. Hypothetical proteins localized by endogenous tagging in this study are highlighted in blue. Parentheses indicate references specific to supplemental information. Download Table S1, PDF file, 0.1 MB. Copyright © 2023 Back et al. 2023 Back et al. https://creativecommons.org/licenses/by/4.0/ This content is distributed under the terms of the Creative Commons Attribution 4.0 International license.

10.1128/mbio.03042-22.9 TABLE S2 List of top hits identified by mass spectrometry of IMC29-BioID cytoskeletal fraction. Proteins were ranked by NSAF. Proteins present in the RH-Control were not excluded but only included in this list if the ratio of IMC29:RH >2. Gene IDs and descriptions are from ToxoDB. Hypothetical proteins localized by endogenous tagging in this study are highlighted in blue. Parentheses indicate references specific to supplemental information. Download Table S2, PDF file, 0.1 MB. Copyright © 2023 Back et al. 2023 Back et al. https://creativecommons.org/licenses/by/4.0/ This content is distributed under the terms of the Creative Commons Attribution 4.0 International license.

**FIG 7:**
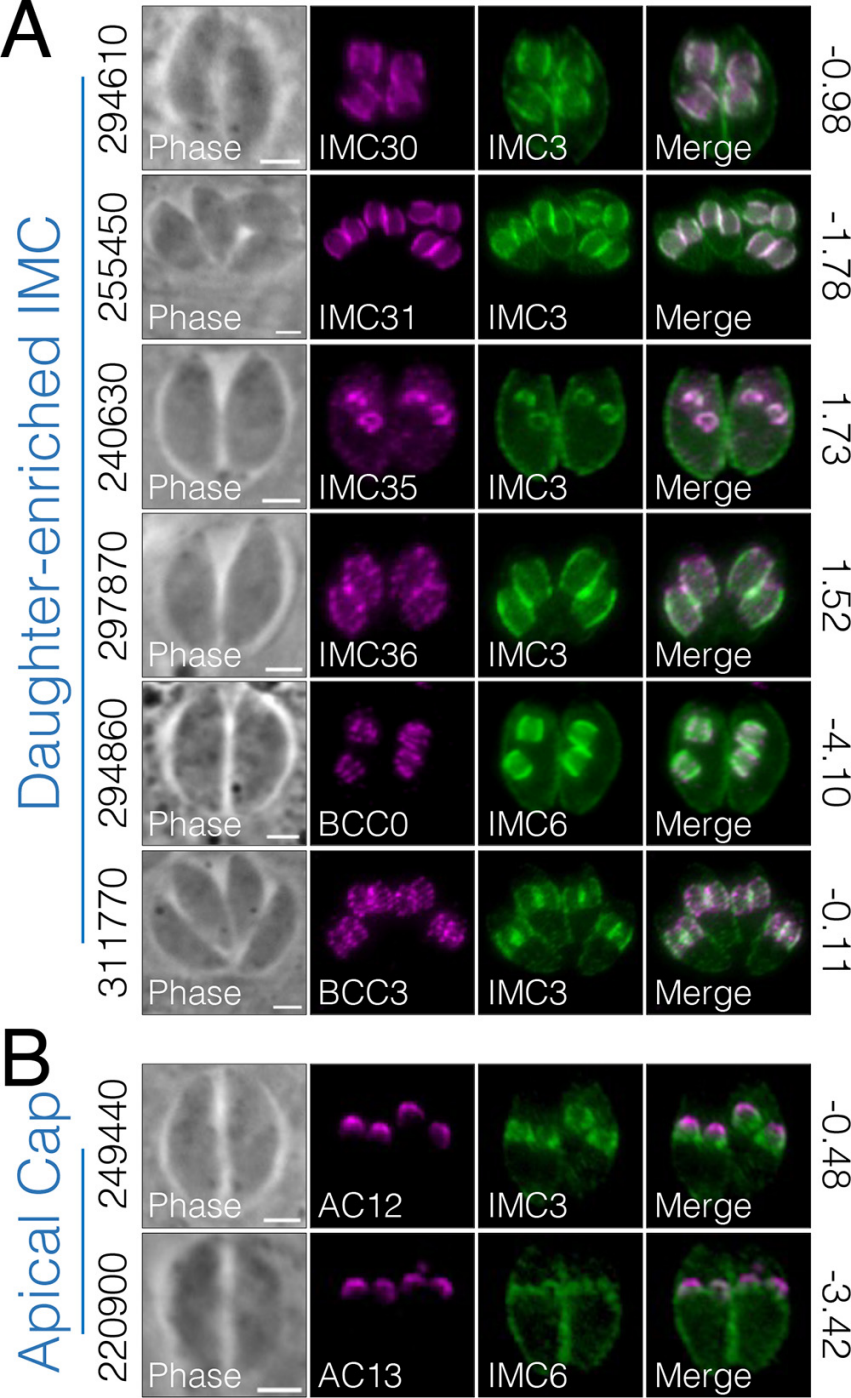
IMC29 BioID reveals new components of the daughter-enriched IMC proteome. (A) IFAs showing six novel IMC proteins that localize to the body portion of the daughter IMC. (B) Two novel IMC proteins localize to the apical cap portion of the daughter IMC. For all panels, gene numbers are shown on the left side of each panel and the GWCS phenotype scores are shown on the right. Novel IMC proteins were designated new names as displayed in the IFA label. For BCC0 (294860): magenta, rat anti-OLLAS; green, rabbit-IMC6. For all other IFAs: magenta, mouse anti-HA; green, rabbit anti-IMC3 or anti-IMC6. All scale bars are 2 μm.

We were able to identify a total of 21 novel IMC proteins from these data sets. Six of these are enriched in or exclusively localize to the daughter IMC, further establishing the daughter-enriched IMC as a distinct subcompartment with its own cohort of proteins (Fig. 7A). IMC30 (TgGT1_294610), IMC31 (TgGT1_255450), and IMC36 (TgGT1_297870) are restricted to the IMC body of daughter buds, similar to the localization of IMC29. IMC35 (TgGT1_240630) is a Unc51-like kinase (ULK) that is enriched in the daughter buds with faint staining in the maternal cytoplasm. We additionally localized TgGT1_294860 and TgGT1_311770, which were also enriched in the daughter buds (Fig. 7A). While this study was in revision, Engelberg et al. (38) categorized both of these proteins as basal complex components, so we adopted their nomenclature (TgGT1_294860 is BCC0 and TgGT1_311770 is BCC3). The authors noted that BCC0 and BCC3 exhibit dynamic localization during division. In agreement with this, our data localized these proteins at the IMC body of daughter buds, with fainter and more spot-like staining compared to the other daughter bud proteins (Fig. 7A). We then wanted to determine whether these novel daughter IMC proteins are dependent on IMC29 for localization since they resemble its localization. Thus, we endogenously tagged IMC30, IMC31, IMC35, IMC36, BCC0, and BCC3 in Δ*imc29*^PC^ parasites and performed IFAs (Fig. S7A-F). All six proteins remained intact in the absence of IMC29, indicating that none of them require IMC29 to target properly to the daughter IMC. Finally, we also identified two proteins that localize to the apical cap of daughter buds and thus named them AC12 (TgGT1_249440) and AC13 (TgGT1_220900) (Fig. 7B).

10.1128/mbio.03042-22.7 FIG S7 Novel daughter IMC proteins are not dependent on IMC29 for localization. (A–H) IFAs show the localization of each novel daughter-enriched protein in Δ*imc29* parasites. (A–D, F) Magenta, mouse anti-HA; green, rabbit anti-IMC6. (E, G–H) Magenta, rat anti-OLLAS; green, rabbit anti-IMC6. (I, J) IFAs show the partial colocalization of IAP2 and IAP3 with ATrx1, which is unaffected by the absence of IMC29. Magenta, rat anti-OLLAS; green, mouse anti-ATrx1; blue, rabbit anti-IMC6. All scale bars are 2 μm. Download FIG S7, JPG file, 0.9 MB. Copyright © 2023 Back et al. 2023 Back et al. https://creativecommons.org/licenses/by/4.0/ This content is distributed under the terms of the Creative Commons Attribution 4.0 International license.

### Identification of proteins with unique IMC-associated localization.

**FIG 8:**
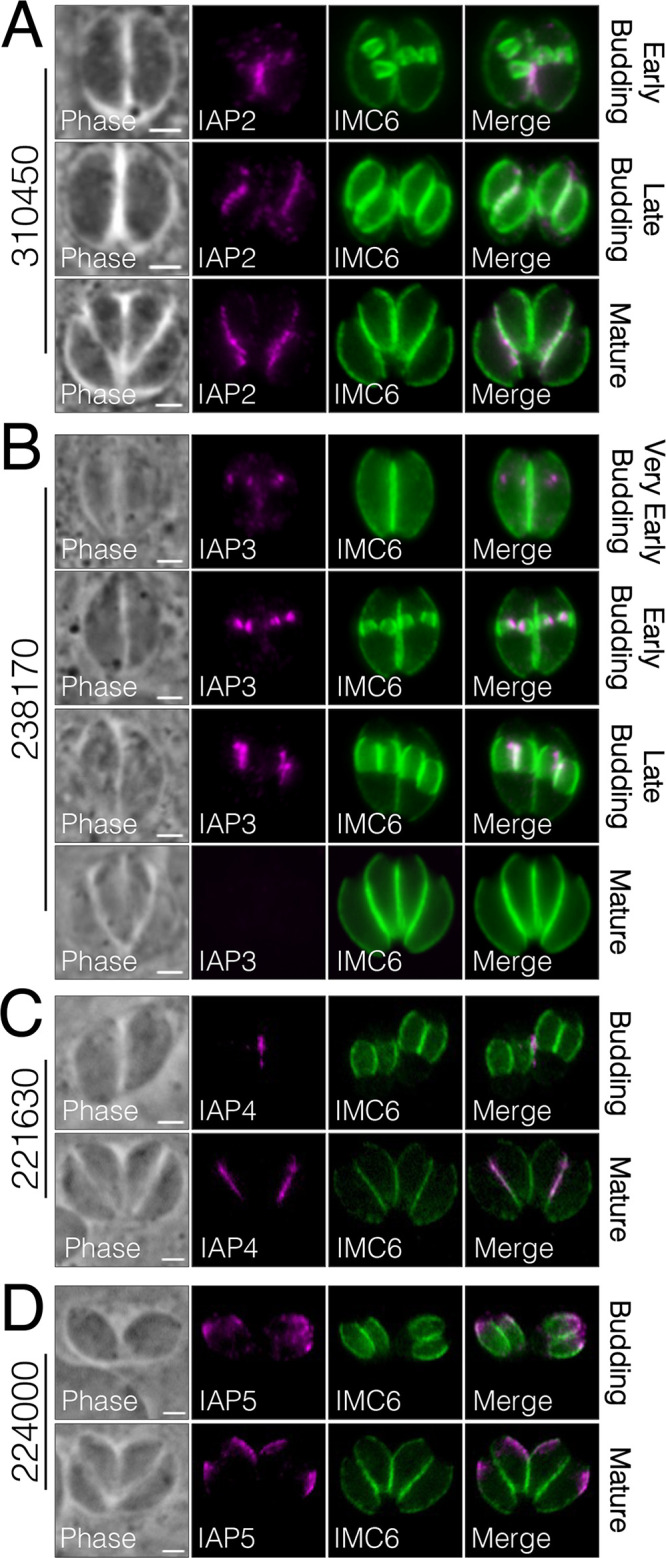
Novel proteins reveal a unique IMC-associated region. (A to C) IFAs display three proteins that localize to the interface between two parasites (310450, IAP2; 238170, IAP3; 221630, IAP4). A and B were stained with magenta, rat anti-OLLAS; green, rabbit anti-IMC6. C was stained with magenta, mouse anti-HA; green, rabbit anti-IMC6. (D) IAP5 (224000) localizes to the opposite face of the IMC in both mature parasites and daughter buds. Magenta, mouse anti-HA; green, rabbit anti-IMC6. For all panels, gene numbers are shown on the left side of each panel, and new names for IMC proteins are designated in the IFA label. All scale bars are 2 μm.

The IMC is categorized into distinct regions, including the apical cap, body, and maternal or daughter buds, based on the compartment-specific localization of IMC proteins. From our IMC29-BioID, we were intrigued to identify four proteins that localize to novel regions of the parasite that appear to be associated with the IMC (Fig. 8). TgGT1_310450, TgGT1_238170, and TgGT1_221630 localize to the peripheral interface between two intracellular parasites (Fig. 8A to C). While TgGT1_310450 is expressed in both mature parasites and daughter buds, TgGT1_238170 is only expressed in daughter buds, and TgGT1_221630 is only expressed in mature parasites. In contrast, TgGT1_224000 localizes to the opposite face of the IMC in both daughter buds and recently divided mature parasites (Fig. 8D). Since these proteins appear to be in distinct regions of the IMC, we denoted them as IMC-associated proteins (IAPs). TgGT1_310450 is IAP2, TgGT1_238170 is IAP3, TgGT1_221630 is IAP4, and TgGT1_224000 is IAP5 (note that a protein named IAP1 was previously localized to the basal region of the IMC) (39).

**FIG 9:**
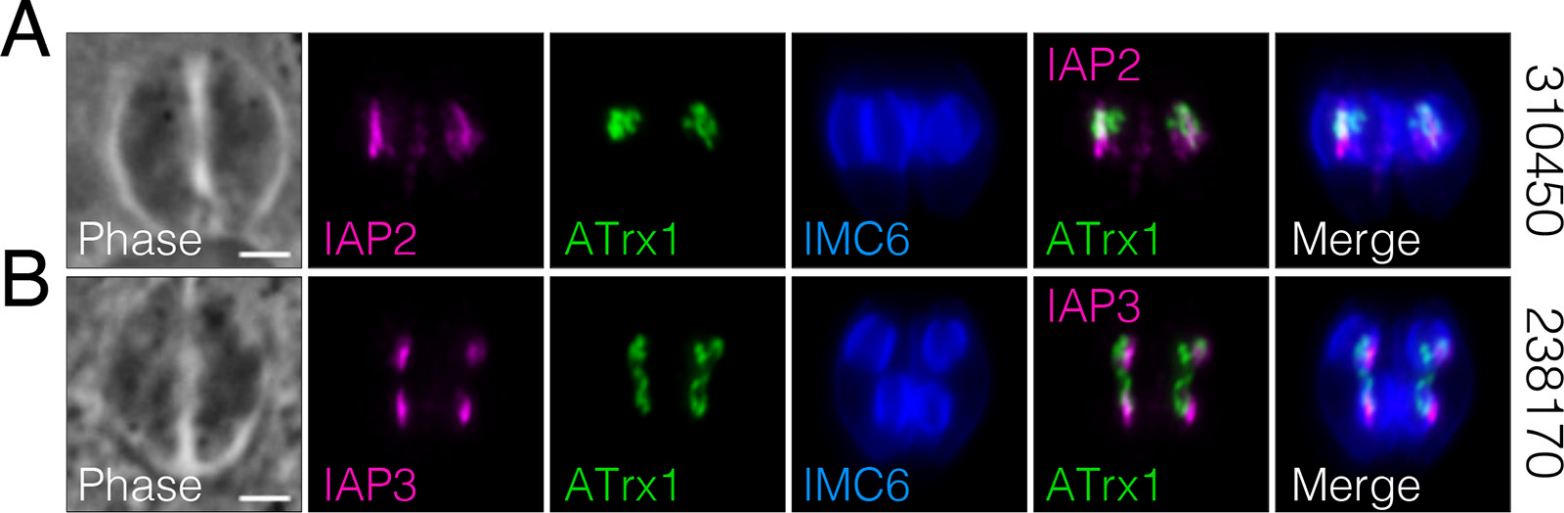
IAP2 and IAP3 partially colocalize with the apicoplast. (A and B) IFAs show regions of partial colocalization between IAP2/IAP3 and the apicoplast. Magenta, rat anti-OLLAS; green, mouse anti-ATrx1 (apicoplast); blue, rabbit anti-IMC6. Gene numbers are on the right side of each panel. Scale bars are 2 μm.

The daughter localization of IAP2 and IAP3 was reminiscent of dividing apicoplasts, which elongate and attach to one side of forming daughter buds during endodyogeny (40). Thus, we costained IAP2 and IAP3 with the apicoplast marker ATrx1 in budding parasites, which showed partial colocalization (Fig. 9). This suggests that the IMC-associated region may be a site at which the apicoplast is tethered to the daughter buds during division. We then wanted to determine if the daughter-enriched ones are dependent on IMC29 for localization, so we endogenously tagged IAP2 and IAP3 in Δ*imc29*^PC^ parasites. Like the other daughter IMC proteins, IFAs showed both proteins targeting properly to the daughter IMC, indicating that neither requires IMC29 for localization (Fig. S7G to J).

**FIG 10:**
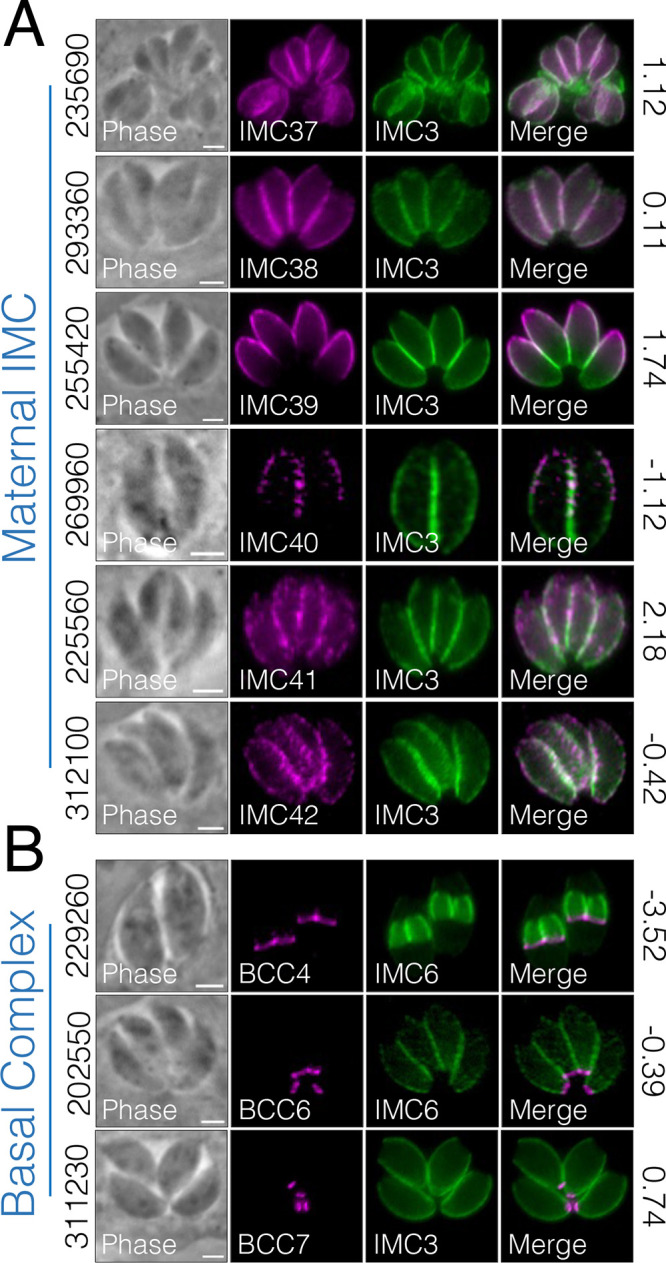
Additional maternal and basal IMC proteins identified by BioID. (A) IFAs show six proteins that localize primarily to the maternal IMC. Magenta, mouse anti-HA; green, rabbit anti-IMC3. New IMC designations are displayed in the IFA label. (B) Three proteins localize to the basal complex of maternal (311230, 202550) or daughter (229260) parasites. Magenta, mouse anti-HA; green, rabbit anti-IMC3 or anti-IMC6. For all panels, gene numbers are shown on the left side of each panel while the GWCS fitness scores are shown on the right. All scale bars are 2 μm.

We also found IMC proteins that localize to the maternal IMC and to the basal complex (Fig. 10). IMC37 (TgGT1_235690) and IMC38 (TgGT1_293360) reside in the maternal IMC, excluded from the apical and basal ends (Fig. 10A). In contrast, IMC39 (TgGT1_255420) localizes to the apical and medial regions but is excluded from the lower third of the maternal IMC. Interestingly, IMC40 (TgGT1_269960), IMC41 (TgGT1_225560), and IMC42 (TgGT1_312100) all exhibit punctate staining along the body of the parasite. We also found three new basal complex proteins: TgGT1_229260, which resides solely in the base of daughter buds, as well as TgGT1_202550 and TgGT1_311230, which reside in the base of maternal parasites (Fig. 10B). While our paper was in revision, these proteins were also localized to the basal IMC by Engelberg et al. (38). Thus, we used the nomenclature of this study, which named TgGT1_229260 as BCC4, TgGT1_202550 as BCC6 and TgGT1_311230 as BCC7. Together, our IMC29-BioID experiments proved valuable in the discovery of novel IMC components to known compartments as well as the identification of a new subregion of the IMC.

## DISCUSSION

In this study, we demonstrate that IMC29 is a key component of early daughter buds, joining a group of other important or essential IMC proteins expressed during the earliest stages of replication. Among these, IMC32 is most like IMC29, as they both localize along the body of early daughter buds and cause severe defects in IMC formation, though IMC32 is essential (22). Another similar component is FBXO1, which also causes disruptions to IMC development, but unlike IMC29 and IMC32, localizes to the apical cap of daughter buds (21). Still other early daughter proteins include the cytoskeletal complex AC9:AC10:ERK7, which is expressed in both the maternal and daughter apical caps (9, 10, 18, 37, 41). However, this complex was recently shown to be dispensable for replication and is instead essential for governing parasite invasion and egress by stabilizing the apical complex. Nonetheless, these studies provide compelling evidence that the earliest expressed daughter IMC proteins tend to be the most critical for IMC function and parasite fitness.

Consistent with its daughter IMC localization, the role of IMC29 is primarily restricted to parasite replication (Fig. 3). Specifically, we observed several phenotypes that were characteristic of disruptions to the IMC. The most striking observation was that the vast majority of Δ*imc29* vacuoles lacked division synchrony, suggesting that Δ*imc29* parasites have lost the ability to properly regulate daughter bud initiation. Similar loss of synchrony has been seen in the depletion of either TgUNC and TgMyoI (42). However, in contrast to IMC29, which likely plays a scaffolding role for daughter bud formation, these myosin proteins are implicated in regulating the cell-cell communication between intravacuolar parasites. Another study found that disrupting the alveolin proteins IMC14 or IMC15 causes division asynchrony and abnormal number of daughter buds, though neither knockout caused growth defects (24). In addition to asynchronous division, many Δ*imc29* parasites contained >2 daughter buds per round of division, which has also been reported in other studies. The most similar of these is Δ*isp2* parasites, exhibiting a high frequency of multiple daughters per maternal cell and resulting in significant growth defects, though unlike IMC29, ISP2 is present in both daughter and mature parasites (23). Other groups have also shown that a similar multidaughter phenotype is observed upon a shortage of lipid and membrane components (43–45). Thus, loss of IMC29 could also disrupt the process of recruiting lipid and membrane precursors that are required to build the IMC, though this requires further experimental validation.

The partial compensation that we observed in Δ*imc29*^PC^ parasites appears to be a fairly common phenomenon in T. gondii. Notably, disrupting the membrane-associated protein ISP2 initially caused severe growth defects that recovered after passaging for 2 months (23). In addition, disrupting the AMA1-RON2 pair revealed that AMA2 as well as a distinct homologue pair (AMA4-RON2_L1_) provided functional redundancy for structuring the moving junction during parasite invasion (46). Moreover, deleting components of the MyoC-glideosome were shown to be compensated by counterparts of the MyoA-glideosome (39). In contrast to these proteins, however, IMC29 does not have obvious orthologues that may be responsible for compensation. It is possible that a more distantly related IMC protein may provide redundant functions, but additional studies are needed to characterize this genetic plasticity.

Many IMC proteins such as HSP20, the ISPs, and IMC32 are anchored to the membrane via palmitoylation, myristoylation, or both (22, 23, 47). Thus, we interrogated the only predicted palmitoylated cysteine of IMC29 and surprisingly found that palmitoylation at this site is dispensable for localization and function (Fig. 5A). Thus, IMC29 represents one of the first membrane-associated IMC proteins in which its predicted acylation site is dispensable for localization. Nonetheless, as palmitoylation is strongly predicted, it is still possible that IMC29 is palmitoylated for membrane attachment but additionally tethered via other IMC proteins. Such tethering may be mediated through CC domains, which often facilitate protein-protein interactions and are abundantly represented in IMC proteins. Previous work has shown that while the IMC sutures-associated protein ISAP1 does not depend on CCs for localization or function (48), other IMC proteins such as ILP1 and IMC32 require their CCs for localization, interaction with associated proteins, or proper function (22, 49).

Thus, we evaluated the predicted CC domains of IMC29 and found that the N-terminal half of the protein containing four predicted CC domains is important for localization while, a short helical region in the C terminus of the protein is critical for function (Fig. 5 and 6). Removing half of the protein containing the four predicted CC domains (IMC29^Δ2-500^) still somewhat localized IMC29 to the daughter buds, indicating that IMC29 may require multiple independent interactions to fully target to the daughter IMC. While we cannot exclude the possibility that localization changes are merely due to protein misfolding, the fact that all N-terminal deletion constructs fully rescue the plaque defects mitigates this possibility (Fig. S5B). Together, these IMC29 deletion analyses identify critical regions for localization and function and corroborate the importance of CC domains in IMC proteins.

Aside from IMC29, there are only a few known proteins that localize to the earliest stages of the daughter bud IMC (e.g., IMC32, FBXO1). Thus, we wanted to discover additional daughter-enriched IMC proteins, including those that may interact with IMC29. We unfortunately could not rely on hyperLOPIT to select candidate daughter IMC proteins because many daughter proteins are incorrectly categorized, likely due to the subcellular fractionation being performed using extracellular parasites which lack daughter buds (50). Thus, we used proximity labeling to identify a suite of novel IMC proteins restricted to the daughter IMC, displaying the power of BioID in labeling such a spatially and temporally specific subcompartment (Fig. 7). The novel proteins IMC30, IMC31, IMC35, and IMC36 all localize to the body portion of the daughter IMC and most resemble the localization of IMC29. In addition, BCC0 and BCC3 were previously shown to display dynamic localization that is not exclusive to the basal complex but exhibit additional localizations in the body of daughter buds, which is consistent with our results that BCC0 and BCC3 partially colocalize with IMC29 (38). The data set also revealed two proteins (AC12 and AC13) that localize specifically to the apical cap of daughter buds. Due to their apical localization and enrichment in the daughter IMC, it is possible that AC12 and AC13 work in conjunction with FBXO1 to build the nascent apical cap, an intriguing question for future studies. Finally, many of these new daughter IMC proteins were assigned dispensable GWCS scores, suggesting that the daughter subcompartment may contain many proteins with redundant functions (25). Together, these novel IMC components represent new areas of study with potential to reveal deeper mechanisms that drive the construction of the daughter cell scaffold.

In addition to the daughter-enriched IMC proteins, we were also able to identify several maternal IMC proteins as well as previously reported BCC proteins (Fig. 10) (38). These could be the result of low levels of the bait protein in the maternal IMC, the promiscuous activity of BirA*, or the long labeling time. This is evident in the streptavidin staining, in which the daughter IMC subcompartment is enriched but some staining is present in the maternal parasites as well (Fig. S6C). Importantly, we discovered four proteins with unique IMC-associated localizations to the interface region between two parasites or to the outward-facing region (Fig. 8). For IAP2 and IAP3, we found that both proteins partially colocalize with the apicoplast during division, suggesting a role in tethering the dividing apicoplasts to the IMC during division, as previously proposed (40). Interestingly, IAP2 is also annotated as a putative myosin heavy chain on ToxoDB. Aside from the canonical roles of myosin proteins in parasite motility (17), *Toxoplasma* myosins have also been shown to be important for proper segregation of daughter cells (51). Thus, consistent with its unique localization, IAP2 may represent another myosin protein implicated in the cytokinesis stages of division. In addition, a similar localization was reported for TgArk3, an aurora kinase that is found at the duplicated centrosomes during S phase but relocalizes to the interface between growing daughter buds during cytokinesis (52, 53). Since TgArk3 was also shown to be important for parasite replication, future studies to determine whether IAP2 and IAP3 are substrates of this kinase would provide valuable mechanistic insights into this subregion. As this region appears to be distinct from other compartments of the IMC, deeper characterization of the IAPs promises to reveal exciting new IMC functions. Together, we reveal a unique subregion of the parasite and identify a large array of novel IMC proteins that expand our understanding of *Toxoplasma* biology.

## MATERIALS AND METHODS

### T. gondii and host cell culture.

Parental T. gondii RHΔ*hxgprt* (wild-type) and subsequent strains were grown on confluent monolayers of human foreskin fibroblasts (HFF) (BJ, ATCC, Manassas, VA) at 37°C and 5% CO_2_ in Dulbecco’s modified eagle medium (DMEM) supplemented with 5% fetal bovine serum (Gibco), 5% Cosmic calf serum (HyClone), and 1× penicillin-streptomycin-l-glutamine (Gibco). Constructs containing selectable markers were selected using 1 μM pyrimethamine (dihydrofolate reductase-thymidylate synthase [DHFR-TS]), 50 μg/mL mycophenolic acid-xanthine (HXGPRT), or 40 μM chloramphenicol (CAT) (54–56). Homologous recombination to the UPRT locus was negatively selected using 5 μM 5-fluorodeoxyuridine (FUDR) (57).

### Antibodies.

The HA epitope was detected with mouse monoclonal antibody (MAb) HA.11 (BioLegend; 901515), rabbit polyclonal antibody (pAb) anti-HA (Invitrogen; PI715500), or mouse MAb HA.11 conjugated to Alexa Fluor-594 (BioLegend 901511). The Ty1 epitope was detected with mouse MAb BB2 (58). The c-Myc epitope was detected with mouse MAb 9E10 (59). The V5 epitope was detected with mouse MAb anti-V5 (Invitrogen; R96025). The smOLLAS epitope was detected with rat MAb anti-OLLAS (60). *Toxoplasma*-specific antibodies include pAb rabbit anti-IMC6 (49), mouse MAb anti-ISP1 (23), pAb rabbit anti-IMC3 (61), pAb rabbit anti-IMC12 (41), pAb rabbit anti-ROP13 (62), MAb mouse anti-F_1_β subunit (5F4) (39), MAb mouse anti-ATrx1 (11G8) (63), pAb guinea pig anti-NHE3 (64), MAb mouse anti-MIC2 (65), MAb mouse anti-ROP7 (66).

### Endogenous epitope tagging and knockout.

For C-terminal endogenous tagging, a pU6-Universal plasmid containing a protospacer against the 3′ untranslated region (UTR) of the target protein (IMC29, AC9, FBXO1, IMC32, as well as all novel IMC proteins) approximately 100 bp downstream of the stop codon was generated, as described previously (67). A homology-directed repair (HDR) template was PCR amplified using the Δ*ku80*-dependent LIC vector p3xHA.LIC-DHFR, p3xMyc.LIC-DHFR, p2xStrep3xTy.LIC-CAT, p3xV5.LIC-DHFR, and pSMOLLAS.LIC-DHFR, all of which include the epitope tag, 3′ UTR, and a selection cassette (68). The 60-bp primers include 40 bp of homology immediately upstream of the stop codon or 40 bp of homology within the 3′ UTR downstream of the CRISPR/Cas9 cut site. Primers that were used for the pU6-Universal plasmid as well as the HDR template are listed in Table S3. P1 to P4 and P13 to P24 were used to tag proteins for timing comparison and P38 to P121 were used to tag novel IMC proteins.

10.1128/mbio.03042-22.10 TABLE S3 Oligonucleotide primers used in this study. All primer sequences are shown in the 5′ to 3′ orientation. Download Table S3, PDF file, 0.03 MB. Copyright © 2023 Back et al. 2023 Back et al. https://creativecommons.org/licenses/by/4.0/ This content is distributed under the terms of the Creative Commons Attribution 4.0 International license.

For knockout of IMC29, the protospacer was designed to target the coding region of IMC29 (TgGT1_243200), ligated into the pU6-Universal plasmid and prepared similarly to the endogenous tagging constructs (P5 to P6 and P9 to P10). The HDR template included 40 bp of homology immediately upstream of the start codon or 40 bp of homology downstream of the region used for homologous recombination for endogenous tagging (P7 to P8 and P11 to P12). The HDR template was PCR amplified from a pJET vector containing the HXGPRT drug marker driven by the NcGRA7 promoter.

For all tagging and knockout constructs, approximately 50 μg of the sequence-verified pU6-Universal plasmid was precipitated in ethanol, and the PCR-amplified HDR template was purified by phenol-chloroform extraction and precipitated in ethanol. Both constructs were electroporated into the appropriate parasite strain. Transfected cells were allowed to invade a confluent monolayer of HFFs overnight, and the appropriate selection was subsequently applied. Successful tagging was confirmed by IFA, and clonal lines of tagged parasites were obtained through limiting dilution.

### Complementation of Δ*imc29*.

The full coding region of IMC29 was PCR amplified from cDNA using primers P21/P22 and cloned into a UPRT-locus knockout vector (10) using BglII/NotI (all enzymes purchased from NEB). The endogenous promoter was amplified from genomic DNA using primers P23/P24 and inserted with NsiI/BglII upstream of the coding sequence, resulting in UPRTKO-IMC29pro-IMC29^FL^. This complement vector was then linearized with DraIII-HF and transfected into Δ*imc29*^PC^ parasites along with a pU6 that targets the UPRT coding region. Selection was performed with 5 μg/mL 5-fluorodeoxyuridine (FUDR) for replacement of UPRT (57). Potential clones were screened by IFA, and an HA-positive clone was designated IMC29^FL^.

For IMC29 deletion constructs, UPRTKO-IMC29pro-IMC29^FL^ was used as the template to amplify truncations from the IMC29 coding region, using primers P25 to P31. The P22 reverse primer was utilized to amplify each N-terminal truncation. The P21 forward primer was utilized to amplify the two C-terminal truncations. Each insert was cloned into a BglII/NotI-digested UPRTKO-IMC29pro-IMC29^FL^. For the additional C-terminal truncation (Δ1111-1218), the UPRTKO-IMC29pro-IMC29^FL^ plasmid was used as the template using the Q5 mutagenesis kit. Primers P36 and P37 were used for inverse PCR to amplify the entire plasmid except for residues 1111 to 1218. For the phosphorylation mutant construct, phosphorylation sites were annotated from ToxoDB, combining phosphoproteomic data of both TgME49_243200 and TgGT1_243200. These include T42, S47, S50, S52, S68, S69, S73, T80, S205, S206, S526, S528, S736, T778, T779, T795, S797, T811, S813, S815, S817, S844, S846, T977, S1087, T1090, T1097, Y1100, S1105, S1106, T1220, and T1230. The mutated residues were designed in three synthetic gene blocks and ligated together using BglII/SgrAI, SgrAI/BamHI, and BamHI/NotI to generate a full-length IMC29 with all 32 residues mutated simultaneously to alanine. The same processes for linearization, transfection, and selection were followed for all deletion and mutant constructs.

### Immunofluorescence assay and Western blot.

Confluent HFF cells were grown on glass coverslips and infected with T. gondii. After 18 to 36 h, the coverslips were fixed with 3.7% formaldehyde in phosphate-buffered saline (PBS) and processed for immunofluorescence as described (62). Primary antibodies were detected by species-specific secondary antibodies conjugated to Alexa Fluor 488/594 (ThermoFisher). Coverslips were mounted in Vectashield (Vector Labs, Burlingame, CA), viewed with an Axio Imager.Z1 fluorescence microscope (Zeiss), and processed with ZEN 2.3 software (Zeiss).

For Western blot, parasites were lysed in 1× Laemmli sample buffer with 100 mM DTT and boiled at 100°C for 10 min. Lysates were resolved by SDS-PAGE and transferred to nitrocellulose membranes, and proteins were detected with the appropriate primary antibody and corresponding secondary antibody conjugated to horseradish peroxidase. Chemiluminescence was induced using the SuperSignal West Pico substrate (Pierce) and imaged on a ChemiDoc XRS+ (Bio-Rad, Hercules, CA).

### Plaque assay.

HFF monolayers were infected with 200 parasites per well of individual strains and allowed to form plaques for 7 days. Cells were then fixed with ice-cold methanol and stained with crystal violet. The areas of 50 plaques per condition were measured using ZEN software (Zeiss). Graphical and statistical analyses were performed using Prism GraphPad 8.0. Multiple two-tailed *t* tests were used to compare the SD-centered means.

### Invasion and egress assays.

Invasion assays were performed as previously described (69). Briefly, parasites were syringe-lysed through a 27.5-gauge needle, resuspended in Endo buffer and settled onto coverslips with HFF monolayers for 20 min. Endo buffer was then replaced with warm D1 media (DMEM, 20 mM HEPES, 1% fetal bovine serum) and incubated at 37°C for 10 min. Coverslips were fixed and blocked (3% bovine serum albumin [BSA] in PBS), and extracellular parasites were stained with anti-SAG1 antibodies. The samples were then permeabilized (3% BSA, 0.2% Triton X-100 in PBS), and all parasites were stained with the anti-F_1_β ATPase antibody and incubated with appropriate secondary antibodies. Parasites were scored as invaded (SAG1–, F_1_β ATPase+) or not (SAG1+, F_1_β ATPase+) by fluorescence microscopy. These assays were performed in triplicate; at least 10 fields were counted for each replicate with a total of >500 individual parasites per replicate. Significance was determined using multiple two-tailed *t* tests and the Mann-Whitney test.

For egress assays, parasites were grown on a monolayer on coverslips for 24 to 36 h until most vacuoles contained 16 or 32 parasites. Coverslips were washed twice with prewarmed PBS and incubated with A23187 (or DMSO control) diluted in PBS at 37°C for 2 min. Coverslips were then fixed and stained with rabbit anti-IMC12 antibody. Three replicate coverslips were performed, with at least 10 fields counted for a total of ~200 vacuoles for each parasite strain. Significance was determined using two-way ANOVA of SD-centered means and Tukey’s multiple-comparison test.

### Replication defects.

HFF monolayers grown on glass coverslips were infected with parasites at a low multiplicity of infection (MOI). At 24 and 32 h postinfection, coverslips were fixed with 3.7% PFA, processed for immunofluorescence, and labeled with anti-ISP1 and anti-IMC6 to identify parasite number in each vacuole and to characterize any replication defects. To score vacuoles containing >2 daughter buds per division round, we counted >150 vacuoles spread across 15 fields. Vacuoles were categorized as >2 daughter buds if even one dividing parasite within the vacuole exhibited more than two daughter buds. To score asynchronous division, we counted >200 vacuoles over 15 fields. Only vacuoles with 4 or more maternal parasites were considered because synchronicity in smaller vacuoles was more variable. For failed vacuoles, 400 to 1,000 vacuoles were counted over 15 fields, categorized as failed if vacuoles exhibited gross morphological defects by IMC staining. All immunofluorescence assays were performed in triplicate. Significance was determined using multiple two-tailed *t* tests and the Mann-Whitney test.

### Mouse virulence assays.

Large vacuoles of intracellular RHΔ*hxgprt* (wild type), Δ*imc29*, and IMC29^FL^ parasites were syringe-lysed from infected HFF monolayers and resuspended in Opti-MEM medium (Thermo Fisher Scientific) prior to intraperitoneal injection into female C57BL/6 mice (4 mice per parasite strain) at the appropriate dosages. In parallel, the viability of resuspended parasites for injection was confirmed by plaque assays. Mice were monitored for symptoms of infection, weight loss, and survival for 30 days. Survival graphs were generated on Prism GraphPad.

### Affinity capture of biotinylated proteins.

For affinity capture of proteins from whole-cell lysates, HFF monolayers infected with IMC29^BirA^* or control parasites (RHΔ*hpt*Δ*ku80*, wild type) were grown in medium containing 150 μM biotin for 30 h. Intracellular parasites in large vacuoles were collected by manual scraping, washed in PBS, and lysed in RIPA buffer (50 mM Tris [pH 7.5], 150 mM NaCl, 0.1% SDS, 0.5% sodium deoxycholate, 1% NP-40) supplemented with Complete Protease Inhibitor Cocktail (Roche) for 30 min on ice. Lysates were centrifuged for 15 min at 14,000 × *g* to pellet insoluble material, and the supernatant was incubated with Streptavidin Plus UltraLink resin (Pierce) at room temperature for 4 h under gentle agitation. Beads were collected and washed five times in RIPA buffer, followed by three washes in 8 M urea buffer (50 mM Tris-HCl [pH 7.4], 150 mM NaCl). Samples were submitted for on-bead digests and analyzed by mass spectrometry.

To enrich for cytoskeletal proteins, we performed a second BioID experiment by growing parasites expressing IMC29-BirA* or the control line with 150 μM biotin for 30 h. Intracellular parasites were collected, washed in PBS, and lysed in 1% Triton X-100 lysis buffer supplemented with Complete Protease Inhibitor Cocktail (Roche) for 30 min on ice. Lysates were centrifuged for 15 min at 14,000 × *g* to pellet the detergent-insoluble cytoskeletal fraction. The insoluble pellet was then solubilized using 1% SDS buffer (50 mM Tris [pH 7.5], 150 mM NaCl) and sonication, diluted to a final concentration of 0.1% SDS, and incubated with Streptavidin Plus UltraLink resin (Pierce) at room temperature for 4 h under gentle agitation. Beads were collected by centrifugation and washed five times in 0.1% SDS buffer (50 mM Tris-HCl [pH 7.4], 150 mM NaCl), followed by three washes in 8 M urea buffer (50 mM Tris-HCl [pH 7.4], 150 mM NaCl). Samples were submitted for on-bead digests and subsequently analyzed by mass spectrometry.

### Mass spectrometry of biotinylated proteins.

Purified proteins bound to streptavidin beads were reduced, alkylated, and digested by sequential addition of lys-C and trypsin proteases (70, 71). Samples were then desalted using C18 tips (Pierce) and fractionated online using a 75-μm inner-diameter fritted fused silica capillary column with a 5-μm pulled electrospray tip and packed in-house with 25 cm of C18 (Dr. Maisch GmbH) 1.9-μm reversed-phase particles. The gradient was delivered by a 140-minute gradient of increasing acetonitrile and eluted directly into a Thermo Orbitrap Fusion Lumos instrument where MS/MS spectra were acquired by data-dependent acquisition (DDA). Data analysis was performed using ProLuCID and DTASelect2 implemented in Integrated Proteomics Pipeline IP2 (Integrated Proteomics Applications) (72–74). Database searching was performed using a FASTA protein database containing T. gondii GT1-translated open reading frames downloaded from ToxoDB. Protein and peptide identifications were filtered using DTASelect and required a minimum of two unique peptides per protein and a peptide-level false-positive rate of less than 5% as estimated by a decoy database strategy. Candidates were ranked by normalized spectral abundance factor values comparing AC9^BioID^ versus control samples (75).

### Bioinformatic analysis for coiled-coil predictions.

The full IMC29 coding sequence was queried using the NPS@ server (https://prabi.ibcp.fr/htm/site/web/home) and DeepCoil through the MPI Bioinformatics Toolkit (https://toolkit.tuebingen.mpg.de) (28–31). For DeepCoil, a 500-residue-limit prevented the full sequence from being analyzed. Thus, only the N-terminal 500 and C-terminal 500 residues were input as two independent queries. Each analysis produced a probability graph, which was annotated manually using Adobe Illustrator.

### Animal experimentation ethics statement.

Specific details of our protocol were approved by the UCLA Institutional Animal Care and Use Committee, known as the Chancellor’s Animal Research Committee (protocol: 2004-055.). Mice were euthanized when the animals reached a moribund state; euthanasia was performed following AVMA guidelines.
