# Supplementary figures and images for: IMC29 Plays an Important Role in Toxoplasma Endodyogeny and Reveals New Components of the Daughter-Enriched IMC Proteome
Source: mBio. 2023 Jan 9;14(1):e03042-22. doi: 10.1128/mbio.03042-22 (PMC9973257; doi:10.1128/mbio.03042-22)

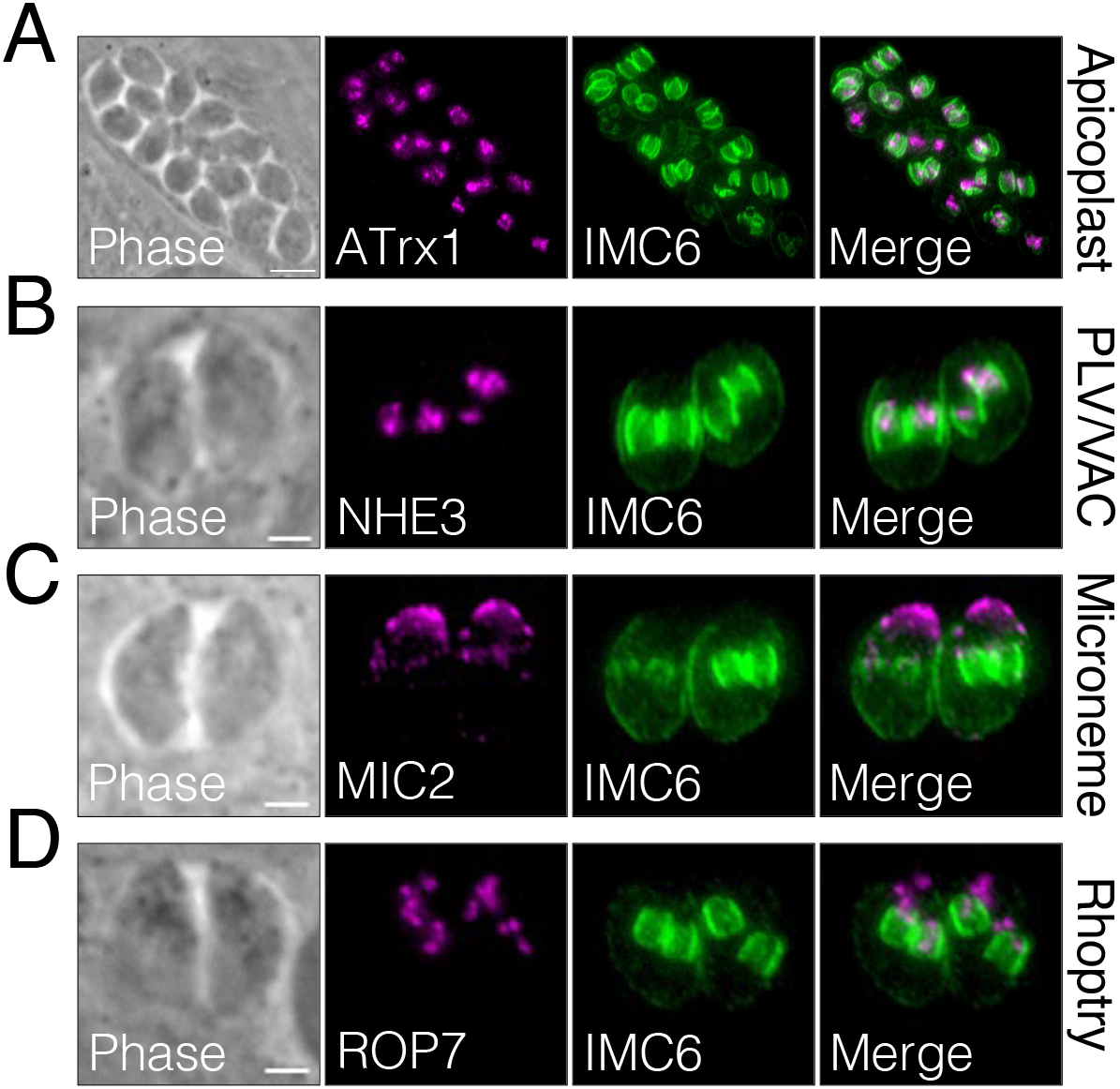

Supplement: FIG S1 [file mbio.03042-22-s0001.tif]

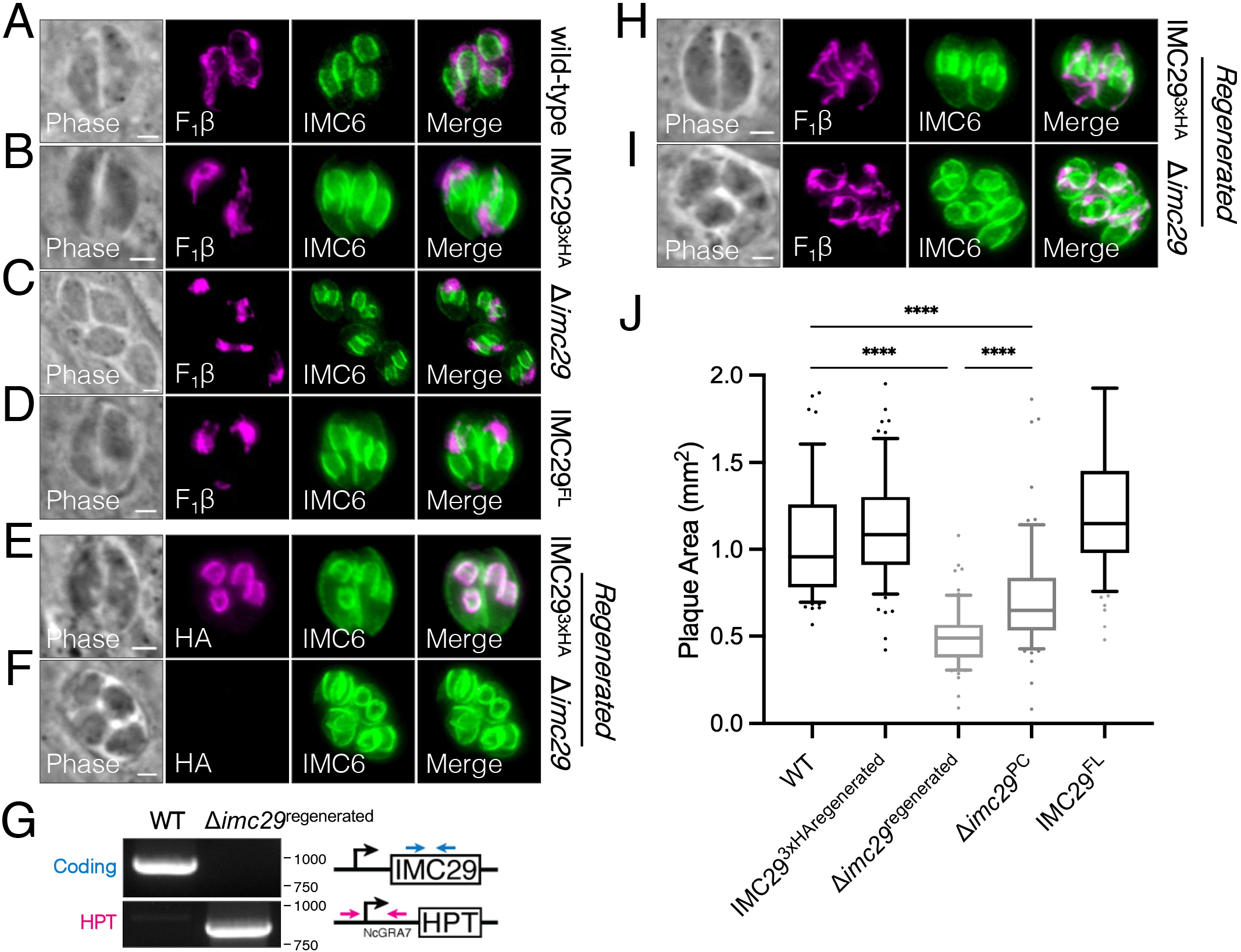

Supplement: FIG S2 [file mbio.03042-22-s0002.jpg]

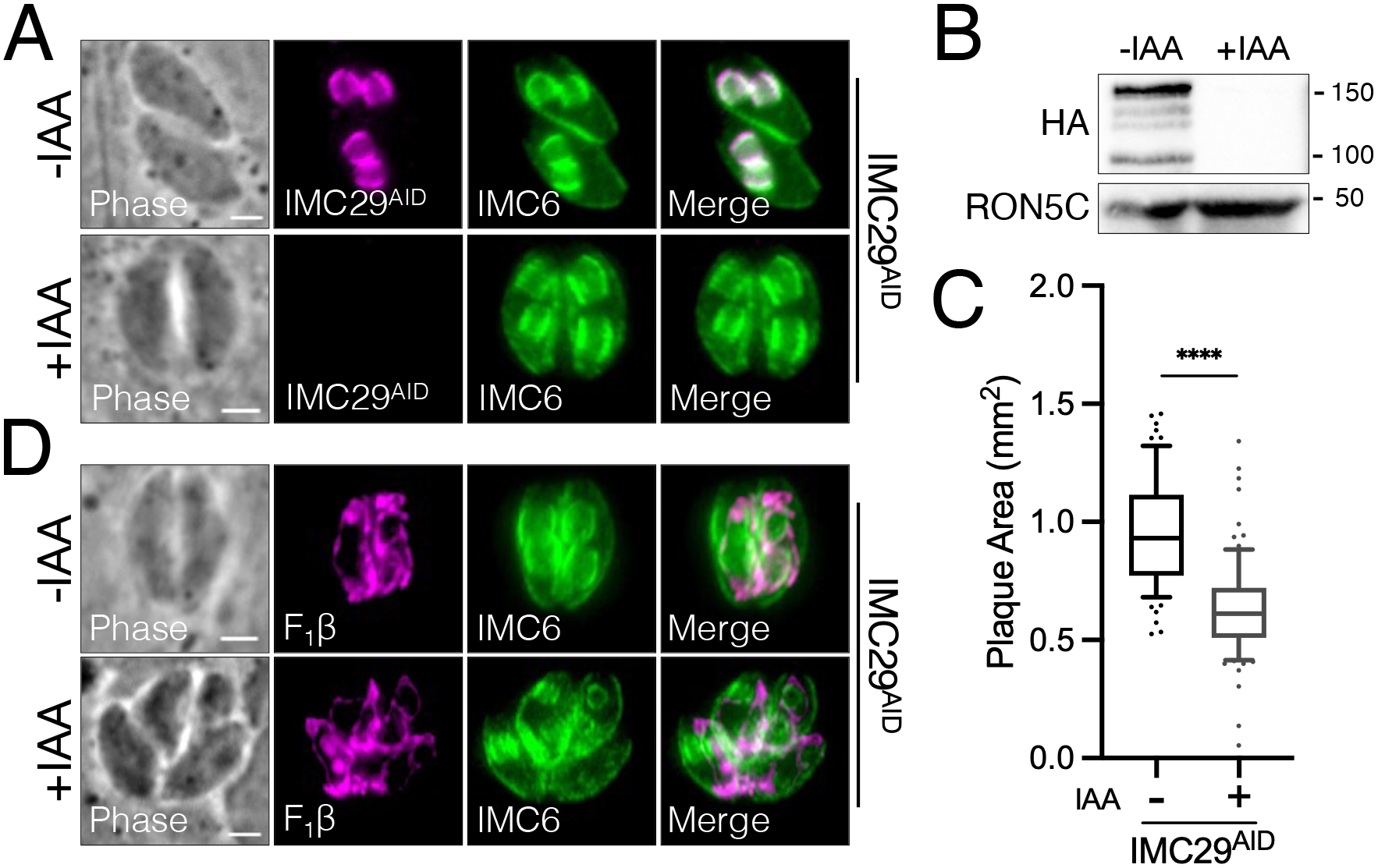

Supplement: FIG S3 [file mbio.03042-22-s0003.tif]

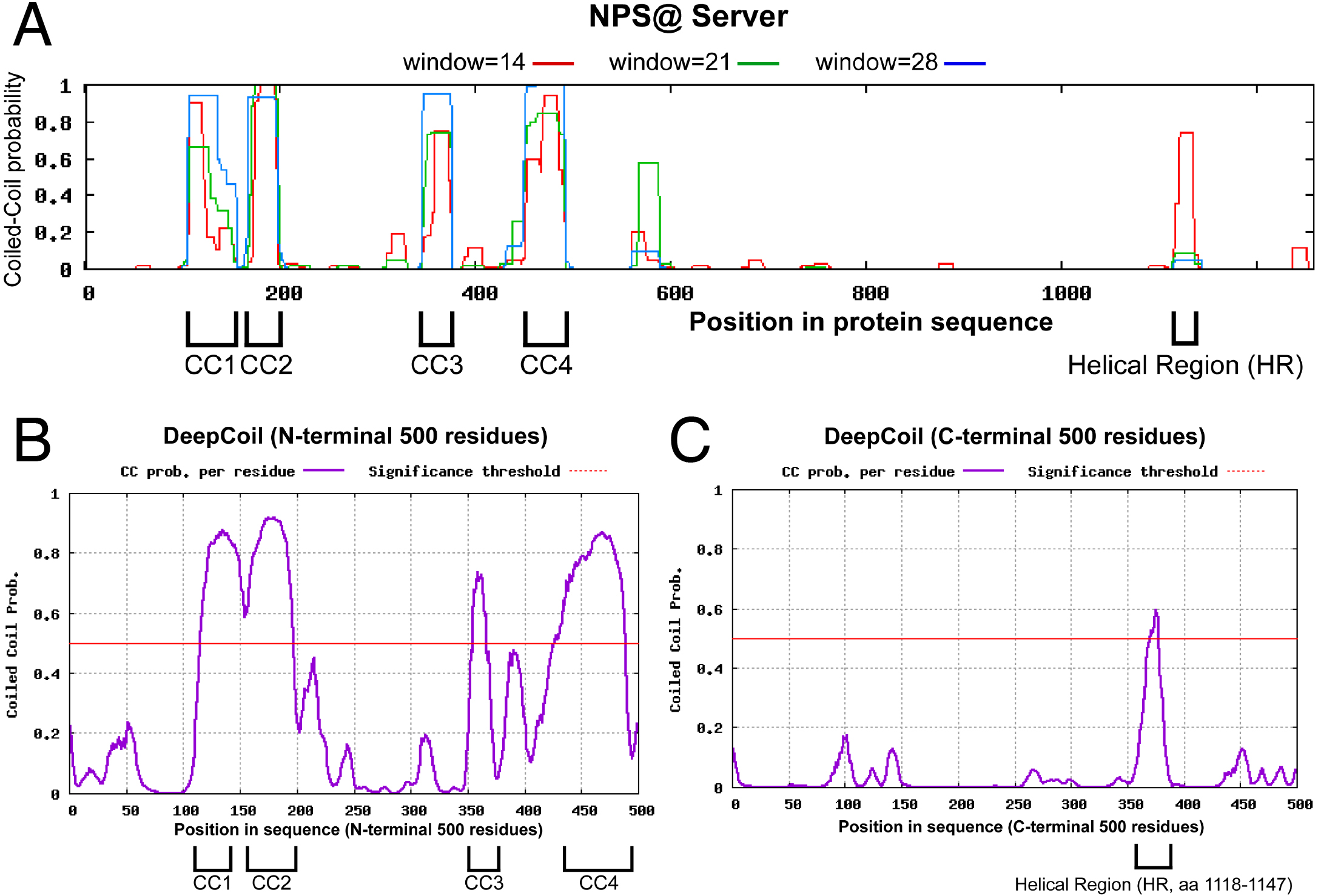

Supplement: FIG S4 [file mbio.03042-22-s0004.jpg]

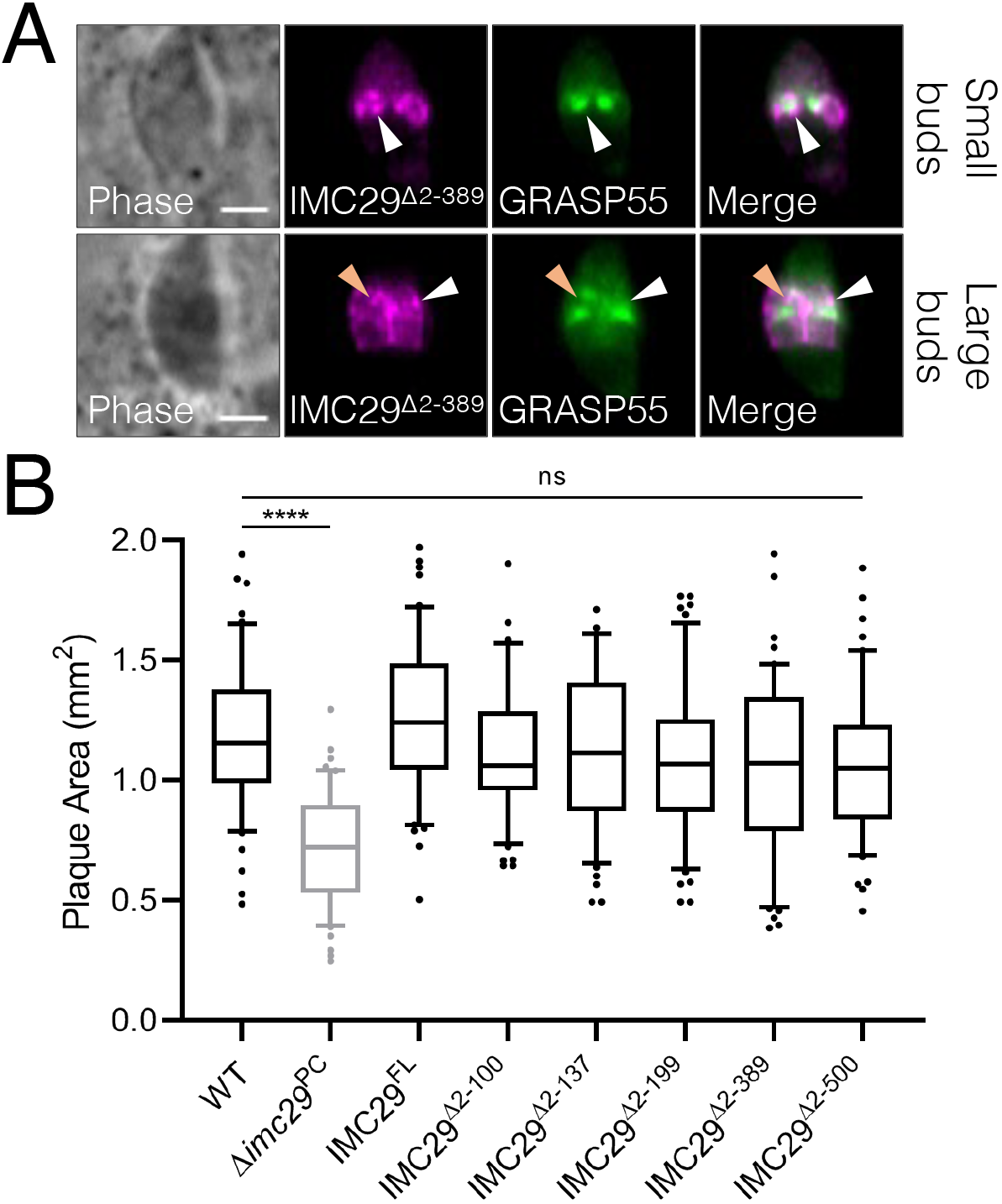

Supplement: FIG S5 [file mbio.03042-22-s0005.tif]

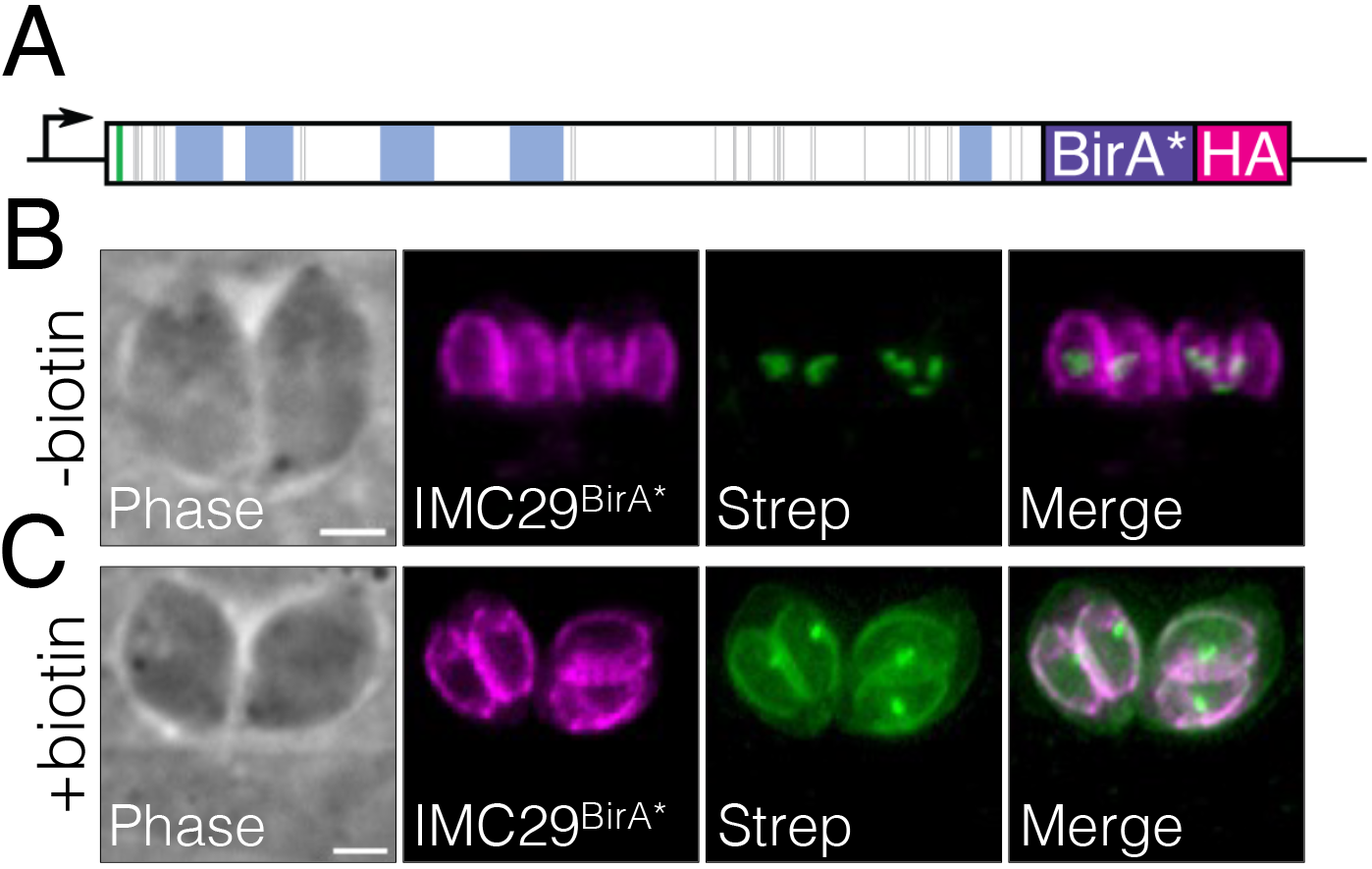

Supplement: FIG S6 [file mbio.03042-22-s0006.tif]

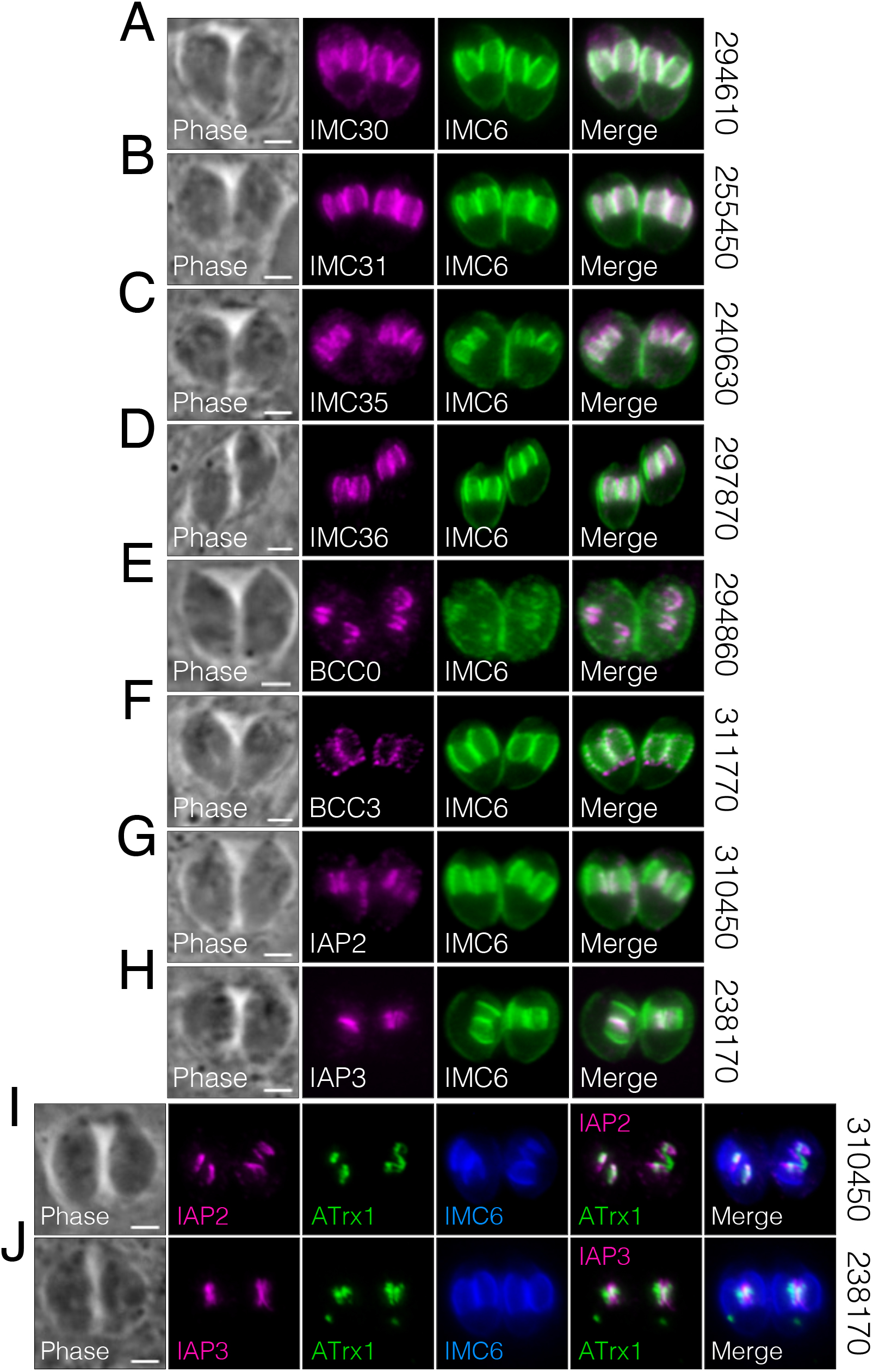

Supplement: FIG S7 [file mbio.03042-22-s0007.jpg]
