# Supplementary material for: IMC29 Plays an Important Role in Toxoplasma Endodyogeny and Reveals New Components of the Daughter-Enriched IMC Proteome
Source: mBio. 2023 Jan 9;14(1):e03042-22. doi: 10.1128/mbio.03042-22 (PMC9973257; doi:10.1128/mbio.03042-22)
Supplement: TABLE S1 [file mbio.03042-22-s0008.pdf]

Table S1. List of top hits identified by mass spectrometry of IMC29-BioID whole cell lysates. Proteins were ranked by NSAF. Proteins present in the RH-Control were not excluded but only included in this list if the ratio of IMC29:RH > 2. Gene IDs and descriptions are from ToxoDB. Hypothetical proteins localized by endogenous tagging in this study are highlighted in blue. Parentheses indicate references specific to supplemental information.

Gene ID	Rank	IMC29 (NSAF[5])	RH-Control (NSAF[5])	Description
TGGT1_231640	1	1183.31015	12.596938	IMC1 (1)
TGGT1_235340	2	885.5741	0	ISC1 (2)
TGGT1_231630	3	815.5314	0	IMC4 (3)
TGGT1_243200	4	683.71524	0	IMC29 (bait)
TGGT1_278975	5	417.5837	0	ICE family protease (caspase) p20 domain-containing protein
TGGT1_226220	6	364.59644	0	IMC9 (4)
TGGT1_230210	7	320.22991	0	IMC10 (4)
TGGT1_294610	8	319.2808	0	IMC30 (this study)
TGGT1_232340	9	297.48487	21.037116	PPM2A (2)
TGGT1_298610	10	267.8377	0	GYF domain-containing protein
TGGT1_294860	11	264.91531	0	BCC0 (5 & this study)
TGGT1_258410	12	241.52019	0	PHIL1 (6)
TGGT1_293730	13	239.40594	43.4536	DHHC14 (7)
TGGT1_212770	14	236.70264	0	IMC34 (8)
TGGT1_216000	15	203.6985	0	IMC3 (9)
TGGT1_255450	16	180.6571	0	IMC31 (this study)
TGGT1_313380	17	169.68115	27.9983	ILP1 (10)
TGGT1_217890	18	169.43382	78.28097	Putative alkyl hydroperoxide reductase
TGGT1_320080	19	168.72487	0	Hypothetical protein
TGGT1_235690	20	167.44047	0	IMC37 (this study)
TGGT1_282070	21	161.51147	161.51147	IMC33 (8)
TGGT1_249180	22	152.43487	62.88143	Bifunctional dihydrofolate reductase-thymidylate synthase
TGGT1_231070	23	151.17918	0	BCC2 (5)
TGGT1_280370	24	148.37147	35.283013	Hypothetical protein
TGGT1_211680	25	148.06573	32.575522	Protein disulfide isomerase
TGGT1_321620	26	139.01453	0	Dynamin-related protein DRPB
TGGT1_203010	27	136.99314	0	Aurora Kinase (11)
TGGT1_220270	28	134.63118	0	IMC6 (4)
TGGT1_268200	29	133.24609	65.11333	RNA recognition motif-containing protein
TGGT1_204160	30	131.20985	0	GYF domain-containing protein
TGGT1_290920	31	123.63236	0	Oxidoreductase, 2OG-Fe(II) oxygenase family protein
TGGT1_305190	32	119.02878	0	CorA family Mg2+ transporter protein
TGGT1_292950	33	115.7869	0	AC10 (12)
TGGT1_225960	34	114.97692	0	STE kinase
TGGT1_304670	35	108.07671	22.696849	IMC23 (2)
TGGT1_239800	36	107.47258	24.826976	TSC2 (13)
TGGT1_230940	37	106.84454	15.111362	Hypothetical protein
TGGT1_243960	38	95.835334	33.689302	Nuclear transport factor 2 (ntf2) domain-containing protein
TGGT1_228150	39	94.21	0	Hypothetical protein
TGGT1_209950	40	93.98781	0	Putative thioredoxin
TGGT1_275670	41	91.18085	0	IMC15 (4)
TGGT1_238895	42	90.35104	0	Hypothetical protein
TGGT1_249870	43	87.1628	0	Localizes to the cytoplasm (2)
TGGT1_260580	44	87.001285	0	Hypothetical protein
TGGT1_311400	45	85.90348	0	WD domain, G-beta repeat-containing protein
TGGT1_295350	46	85.70071	0	Putative nucleoside diphosphate kinase
TGGT1_306220	47	83.637546	0	Hypothetical protein
TGGT1_312270	48	83.022565	38.357673	ROP13 (14)
TGGT1_246950	49	80.818427	0	AC9 (13)
TGGT1_291180	50	80.65225	19.911287	Vacuolar protein sorting 13 (VPS13)
TGGT1_293690	51	80.50673	0	Profilin PRF
TGGT1_231850	52	80.26351	0	PP2C
TGGT1_312590	53	78.801083	0	CLASP family microtubule associated protein
TGGT1_253470	54	75.09579	0	IMC13 (4)
TGGT1_240630	55	73.05986	0	IMC35 (this study)
TGGT1_214575	56	72.193536	0	Hypothetical protein
TGGT1_223000	57	72.193536	0	Dynein light chain DLC
TGGT1_230160	58	70.15991	0	Localizes to the IMC (15)
TGGT1_245470	59	70.01903	0	Mitotic checkpoint protein, BUB3 family protein
TGGT1_231440	60	67.55117	0	LsmAD domain-containing protein
TGGT1_297720	61	65.22231	28.250332	trehalose-phosphatase
TGGT1_272660	62	64.358575	0	Localizes to the endoplasmic reticulum (15)

TGGT1_311770	63	62.386895	0	BCC3 (5 & this study)
TGGT1_251810	64	61.880174	0	Putative translation initiation factor eIF-5A
TGGT1_263090	65	61.68859	23.750881	14-3-3 protein (Weidner et al. 2016)
TGGT1_210345	66	61.4982	0	Hypothetical protein
TGGT1_260600	67	61.42481	25.175084	PUF1
TGGT1_229930	68	61.12091	0	p25-alpha family protein
TGGT1_214880	69	60.639466	0	AC4 (2)
TGGT1_243430	70	59.51439	5.499308	OTU family cysteine protease
TGGT1_255880	71	59.07578	0	Co-localizes with MORN1 (16)
TGGT1_249440	72	59.03827	0	AC12 (this study)
TGGT1_259660	73	58.673194	0	orotate phosphoribosyltransferase
TGGT1_258470	74	58.4666	0	IMC24 (2)
TGGT1_308860	75	55.85616	17.595263	AC3 (2)
TGGT1_313270	76	55.76364	0	Hypothetical protein
TGGT1_207690	77	54.441026	0	Programmed cell death 5 protein
TGGT1_310700	78	54.26312	0	Serine/threonine phosphatase PP1
TGGT1_285650	79	52.82921	0	Hypothetical protein
TGGT1_260540	80	49.836194	0	IMC14 (4)
TGGT1_227600	81	49.56571	0	RPL34
TGGT1_309210	82	49.56571	0	Putative peroxiredoxin 6
TGGT1_315700	83	48.304038	0	Hypothetical protein
TGGT1_291140	84	47.959952	19.4633	CCR4-Not complex component, Not1 protein
TGGT1_263520	85	47.306306	0	SPM1 (17)
TGGT1_311230	86	46.173748	2.499957	BCC7 (3)
TGGT1_310440	87	45.742458	0	MORN1 (18)
TGGT1_297870	88	45.700496	0	IMC36 (this study)
TGGT1_297120	89	43.696087	0	Hypothetical protein
TGGT1_237290	90	43.004492	3.6793932	Hypothetical protein
TGGT1_247350	91	42.575676	0	Thioredoxin domain-containing protein
TGGT1_271930	92	42.575676	0	IMC20 (2)
TGGT1_267060	93	42.304493	0	RPL14
TGGT1_250790	94	42.036743	0	Hypothetical protein
TGGT1_230850	95	41.860118	0	TSC3 (13)
TGGT1_231140	96	41.25345	0	RPS25
TGGT1_207370	97	41.11824	0	Hypothetical protein
TGGT1_214260	98	40.664113	13.419594	Putative alpha-glucan water dikinase 1
TGGT1_239830	99	40.549968	0	TBC domain-containing protein
TGGT1_205320	100	40.44401	15.571452	Hypothetical protein
TGGT1_223790	101	39.731638	0	Cytoplasmic localization (19)
TGGT1_250700	102	39.613154	17.793487	Hypothetical protein
TGGT1_242290	103	39.378292	0	Putative proteasome subunit alpha1
TGGT1_250340	104	39.069445	0	Centrin 2 (20, 21)
TGGT1_275350	105	38.737993	0	TBC domain-containing protein
TGGT1_301410	106	38.577768	0	Hypothetical protein
TGGT1_236040	107	38.347606	0	Fructose-1,6-bisphosphate aldolase
TGGT1_220090	108	38.025604	0	Hypothetical protein
TGGT1_301420	109	37.88102	0	SAS6L (22)
TGGT1_236560	110	37.509188	0	Hypothetical protein
TGGT1_313080	111	36.493436	0	Hypothetical protein
TGGT1_309590	112	36.413406	0	ROP1 (23)
TGGT1_238170	113	36.259054	0	IAP3 (this study)
TGGT1_309870	114	36.009788	0	Localizes to the VAC (24)
TGGT1_268850	115	34.95687	0	Enolase 2
TGGT1_293180	116	34.47304	0	NADP-specific glutamate dehydrogenase
TGGT1_310870	117	33.043805	0	TMCO1 ortholog
TGGT1_283850	118	33.010961	0	Peptidyl-prolyl cis-trans isomerase
TGGT1_291930	119	32.9735	0	RNA recognition motif-containing protein
TGGT1_262640	120	32.55787	0	Cg8 family protein
TGGT1_322000	121	32.55787	0	Myosin-light-chain kinase
TGGT1_248640	122	32.058684	0	Regulator of chromosome condensation (RCC1) repeat-containing protei
TGGT1_214180	123	31.428103	0	ENTH domain-containing protein
TGGT1_311240	124	31.182184	0	Putative DnaJ family chaperone
TGGT1_316680	125	31.058802	2.759545	RNA pseudouridine synthase superfamily protein
TGGT1_248460	126	30.7491	0	Putative ubiquitin
TGGT1_271060	127	30.7491	0	Sec1 family protein
TGGT1_200460	128	30.466996	0	Hypothetical protein
TGGT1_297500	129	30.411198	0	T-complex protein 1 eta subunit
TGGT1_257960	130	30.258796	0	GDP-D-mannose pyrophosphorylase

TGGT1_216900	131	29.985578	0	Cytokine induced apoptosis inhibitor 1 family protein
TGGT1_289050	132	28.757384	0	Putative FIKK kinase
TGGT1_262150	133	28.418482	0	Localizes to the cytoplasm (25)
TGGT1_237210	134	28.41414	0	Tyrosine kinase-like (TKL) protein
TGGT1_221690	135	28.263	10.881609	Hypothetical protein
TGGT1_246740	136	28.09562	0	Hypothetical protein
TGGT1_244500	137	27.953726	0	Tubulin-tyrosine ligase family protein
TGGT1_286590	138	27.906746	0	SPM2 (17)
TGGT1_305610	139	27.616654	0	Hypothetical protein
TGGT1_300260	140	27.543033	9.0894966	Threonyl-tRNA synthetase family protein
TGGT1_202760	141	27.370077	0	Hypothetical protein
TGGT1_309370	142	26.959355	11.3232985	Adaptin N-terminal region domain-containing protein
TGGT1_321680	143	26.781473	0	Hypothetical protein
TGGT1_209420	144	26.567222	0	Hypothetical protein
TGGT1_272350	145	26.567222	0	mRNA export protein (26)
TGGT1_207060	146	25.480072	0	RNR2 (27)
TGGT1_201380	147	24.782855	0	Putative chorismate synthase
TGGT1_258540	148	24.101279	0	Phosphoglycerate mutase family protein
TGGT1_227952	149	23.608311	0	14-3-3 superfamily protein
TGGT1_203540	150	23.5734	0.433139089	Putative RNA binding protein

References for Table S1.

1. Mann T, Beckers C. 2001. Characterization of the subpellicular network, a filamentous membrane skeletal component in the parasite Toxoplasma gondii. Mol Biochem Parasitol 115:257–268.

2. Chen AL, Kim EW, Toh JY, Vashisht AA, Rashoff AQ, Van C, Huang AS, Moon AS, Bell HN, Bentolila LA, Wohlschlegel JA, Bradley PJ. 2015. Novel Components of the Toxoplasma Inner Membrane Complex Revealed by BioID. mBio 6.

3. Hu K, Johnson J, Florens L, Fraunholz M, Suravajjala S, DiLullo C, Yates J, Roos DS, Murray JM. 2006. Cytoskeletal Components of an Invasion Machine—The Apical Complex of Toxoplasma gondii. PLOS Pathogens 2:e13.

4. Anderson-White BR, Ivey FD, Cheng K, Szatanek T, Lorestani A, Beckers CJ, Ferguson DJP, Sahoo N, Gubbels M-J. 2011. A family of intermediate filament-like proteins is sequentially assembled into the cytoskeleton of Toxoplasma gondii. Cellular Microbiology 13:18–31.

5. Engelberg K, Bechtel T, Michaud C, Weerapana E, Gubbels M-J. 2022. Proteomic characterization of the Toxoplasma gondii cytokinesis machinery portrays an expanded hierarchy of its assembly and function. 1. Nat Commun 13:4644.

6. Gilk SD, Raviv Y, Hu K, Murray JM, Beckers CJM, Ward GE. 2006. Identification of PhIL1, a Novel Cytoskeletal Protein of the Toxoplasma gondii Pellicle, through Photosensitized Labeling with 5-[125I]Iodonaphthalene-1-Azide. Eukaryotic Cell 5:1622–1634.

7. Beck JR, Fung C, Straub KW, Coppens I, Vashisht AA, Wohlschlegel JA, Bradley PJ. 2013. A Toxoplasma Palmitoyl Acyl Transferase and the Palmitoylated Armadillo Repeat Protein TgARO Govern Apical Rhoptry Tethering and Reveal a Critical Role for the Rhoptries in Host Cell Invasion but Not Egress. PLOS Pathogens 9:e1003162.

8. Dos Santos Pacheco N, Tosetti N, Krishnan A, Haase R, Maco B, Suarez C, Ren B, Soldati-Favre D. 2021. Revisiting the Role of Toxoplasma gondii ERK7 in the Maintenance and Stability of the Apical Complex. mBio 12:e02057-21.

9. Hu K, Johnson J, Florens L, Fraunholz M, Suravajjala S, DiLullo C, Yates J, Roos DS, Murray JM. 2006. Cytoskeletal Components of an Invasion Machine—The Apical Complex of Toxoplasma gondii. PLOS Pathogens 2:e13.

10. Lorestani A, Ivey FD, Thirugnanam S, Busby MA, Marth GT, Cheeseman IM, Gubbels M-J. 2012. Targeted proteomic dissection of Toxoplasma cytoskeleton sub-compartments using MORN1. Cytoskeleton (Hoboken) 69:1069–1085.

11. Berry L, Chen C-T, Reininger L, Carvalho TG, El Hajj H, Morlon-Guyot J, Bordat Y, Lebrun M, Gubbels M-J, Doerig C, Daher W. 2016. The conserved apicomplexan Aurora kinase TgArk3 is involved in endodyogeny, duplication rate and parasite virulence. Cellular Microbiology 18:1106–1120.

12. Tosetti N, Dos Santos Pacheco N, Bertiaux E, Maco B, Bournonville L, Hamel V, Guichard P, Soldati-Favre D. 2020. Essential function of the alveolin network in the subpellicular microtubules and conoid assembly in Toxoplasma gondii. eLife 9:e56635.

13. Chen AL, Moon AS, Bell HN, Huang AS, Vashisht AA, Toh JY, Lin AH, Nadipuram SM, Kim EW, Choi CP, Wohlschlegel JA, Bradley PJ. 2017. Novel insights into the composition and function of the Toxoplasma IMC sutures. Cellular Microbiology 19:e12678.

14. Bradley PJ, Ward C, Cheng SJ, Alexander DL, Coller S, Coombs GH, Dunn JD, Ferguson DJ, Sanderson SJ, Wastling JM, Boothroyd JC. 2005. Proteomic Analysis of Rhoptry Organelles Reveals Many Novel Constituents for Host-Parasite Interactions in Toxoplasma gondii*. Journal of Biological Chemistry 280:34245–34258.

15. Lorestani A, Ivey FD, Thirugnanam S, Busby MA, Marth GT, Cheeseman IM, Gubbels M-J. 2012. Targeted proteomic dissection of Toxoplasma cytoskeleton sub-compartments using MORN1. Cytoskeleton (Hoboken) 69:1069–1085.

16. Engelberg K, Ivey FD, Lin A, Kono M, Lorestani A, Faugno-Fusci D, Gilberger T-W, White M, Gubbels M-J. 2016. A MORN1-associated HAD phosphatase in the basal complex is essential for Toxoplasma gondii daughter budding. Cellular Microbiology 18:1153–1171.

17. Tran JQ, Li C, Chyan A, Chung L, Morrissette NS. 2012. SPM1 Stabilizes Subpellicular Microtubules in Toxoplasma gondii. Eukaryotic Cell 11:206–216.

18. Gubbels M-J, Vaishnava S, Boot N, Dubremetz J-F, Striepen B. 2006. A MORN-repeat protein is a dynamic component of the Toxoplasma gondii cell division apparatus. Journal of Cell Science 119:2236–2245.

19. Long S, Anthony B, Drewry LL, Sibley LD. 2017. A conserved ankyrin repeat-containing protein regulates conoid stability, motility and cell invasion in Toxoplasma gondii. 1. Nature Communications 8:1–14.

20. Leung JM, Liu J, Wetzel LA, Hu K. 2019. Centrin2 from the human parasite Toxoplasma gondii is required for its invasion and intracellular replication. Journal of Cell Science 132:jcs228791.

21. Lentini G, Dubois DJ, Maco B, Soldati-Favre D, Frénal K. 2019. The roles of Centrin 2 and Dynein Light Chain 8a in apical secretory organelles discharge of Toxoplasma gondii. Traffic 20:583–600.

22. de Leon JC, Scheumann N, Beatty W, Beck JR, Tran JQ, Yau C, Bradley PJ, Gull K, Wickstead B, Morrissette NS. 2013. A SAS-6-like protein suggests that the Toxoplasma conoid complex evolved from flagellar components. Eukaryot Cell 12:1009–1019.

23. Ossorio PN, Schwartzman JD, Boothroyd JC. 1992. A Toxoplasma gondii rhoptry protein associated with host cell penetration has unusual charge asymmetry. Molecular and Biochemical Parasitology 50:1–15.

24. Daher W, Morlon-Guyot J, Sheiner L, Lentini G, Berry L, Tawk L, Dubremetz J-F, Wengelnik K, Striepen B, Lebrun M. 2015. Lipid kinases are essential for apicoplast homeostasis in Toxoplasma gondii. Cellular Microbiology 17:559–578.

25. Heredero-Bermejo I, Varberg JM, Charvat R, Jacobs K, Garbuz T, Sullivan WJ, Arrizabalaga G. 2019. TgDrpC, an atypical dynamin-related protein in Toxoplasma gondii, is associated with vesicular transport factors and parasite division. Mol Microbiol 111:46–64.
26. Courjol F, Mouveaux T, Lesage K, Saliou J-M, Werkmeister E, Bonabaud M, Rohmer M, Slomianny C, Lafont F, Gissot M. 2017. Characterization of a nuclear pore protein sheds light on the roles and composition of the Toxoplasma gondii nuclear pore complex. Cell Mol Life Sci 74:2107–2125.
27. Molina D, Cossio-Pérez R, Rocha-Roa C, Pedraza L, Cortes E, Hernández A, Gómez-Marín JE. 2018. Protein targets of thiazolidinone derivatives in Toxoplasma gondii and insights into their binding to ROP18. BMC Genomics 19:856.
