# Supplementary material for: IMC29 Plays an Important Role in Toxoplasma Endodyogeny and Reveals New Components of the Daughter-Enriched IMC Proteome
Source: mBio. 2023 Jan 9;14(1):e03042-22. doi: 10.1128/mbio.03042-22 (PMC9973257; doi:10.1128/mbio.03042-22)
Supplement: TABLE S2 [file mbio.03042-22-s0009.pdf]

Table S2. List of top hits identified by mass spectrometry of IMC29-BioID cytoskeletal fraction. Proteins were ranked by NSAF. Proteins present in the RH-Control were not excluded but only included in this list if the ratio of IMC29:RH > 2. Gene IDs and descriptions are from ToxoDB. Hypothetical proteins localized by endogenous tagging in this study are highlighted in blue. Parentheses indicate references specific to supplemental information.

Gene ID	Rank	IMC29 (NSAF[5])	RH-Control (NSAF[5])	Description
TGGT1_231640	1	8446.595	768.355	IMC1 (1)
TGGT1_231630	2	5046.7	557.437	IMC4 (2)
TGGT1_316340	3	1512.045	0	IMC22 (3)
TGGT1_313380	4	1229.226	328.417	ILP1 (4)
TGGT1_295360	5	1085.372	134.308	IMC18 (3)
TGGT1_286580	6	1074.066	150.762	IMC17 (3)
TGGT1_230210	7	1066.194	192.828	IMC10 (5)
TGGT1_243200	8	1034.423	243.206	IMC29 (bait)
TGGT1_217510	9	986.434	0	IMC19 (3)
TGGT1_258470	10	970.317	0	IMC24 (3)
TGGT1_216000	11	967.512	200.713	IMC3 (6)
TGGT1_248700	12	939.569	389.832	IMC12 (5)
TGGT1_220270	13	930.98	81.069	IMC6 (5)
TGGT1_259630	14	861.157	0	IMC27 (7)
TGGT1_230850	15	804.068	340.284	TSC3 (7)
TGGT1_222220	16	678.041	209.758	IMC7 (5)
TGGT1_271930	17	654.25	0	IMC20 (3)
TGGT1_294610	18	622.411	0	IMC30 (this study)
TGGT1_250820	19	595.956	217.96	AC2 (3)
TGGT1_304670	20	588.825	0	IMC23 (3)
TGGT1_293360	21	567.017	148.125	IMC38 (this study)
TGGT1_308860	22	514.997	247.669	AC3 (3)
TGGT1_237820	23	478.42	0	ISP2 (8)
TGGT1_246950	24	474.187	0	AC9 (7)
TGGT1_212770	25	472.313	0	IMC34 (9)
TGGT1_219170	26	451.754	0	ISC2 (3)
TGGT1_239400	27	447.786	28.454	IMC28 (7)
TGGT1_231070	28	442.55	130.061	BCC2 (10)
TGGT1_275670	29	415.265	0	IMC15 (5)
TGGT1_264990	30	413.769	0	Hypothetical protein
TGGT1_305930	31	392.55	0	ISC4 (3)
TGGT1_235690	32	385.953	0	IMC37 (this study)
TGGT1_290920	33	379.966	0	Oxidoreductase, 2OG-Fe(II) oxygenase family protein
TGGT1_226220	34	364.511	119.029	IMC9 (5)
TGGT1_288720	35	346.368	0	RPL10
TGGT1_239800	36	341.475	0	TSC2 (7)
TGGT1_260500	37	340.21	0	Putative COPI associated protein
TGGT1_226570	38	330.42	0	Hypothetical protein
TGGT1_255450	39	318.437	57.591	IMC31 (this study)
TGGT1_292950	40	310.136	0	AC10 (11)
TGGT1_253470	41	307.728	90.438	IMC13 (5)
TGGT1_306190	42	293.785	0	Hypothetical protein
TGGT1_278975	43	293.071	0	ICE family protease (caspase) p20 domain-containing protein
TGGT1_282070	44	292.1	40.398	IMC33 (9)
TGGT1_314260	45	287.501	0	Hypothetical protein
TGGT1_315750	46	285.269	0	IMC26 (3)
TGGT1_289600	47	281.424	0	HSP29
TGGT1_316540	48	280.051	0	ISP3 (8)
TGGT1_261570	49	277.345	130.415	RPL7A
TGGT1_272520	50	267.247	0	TSC4 (7)
TGGT1_222400	51	262.598	0	ISC7 (ToxoDB comment)
TGGT1_260820	52	260.957	0	ISP1 (8)
TGGT1_283510	53	243.781	0	IAP1 (12)
TGGT1_312600	54	235.53	0	HSP21
TGGT1_227600	55	228.499	0	RPL34
TGGT1_228470	56	224.041	0	RPL15
TGGT1_293730	57	220.734	0	DHHC14 (13)
TGGT1_255420	58	220.28	0	IMC39 (this study)
TGGT1_313190	59	216.643	0	Rab18/RabC-family small GTPase
TGGT1_235380	60	215.626	0	AC5/TLAP3 (3)
TGGT1_312050	61	214.619	0	Putative small GTPase Rab2
TGGT1_227000	62	207.888	0	Localizes to the cytoplasm (14)
TGGT1_213350	63	204.126	0	RPS15
TGGT1_244320	64	197.541	0	PRPL24

TGGT1_227360	65	196.78	92.531	RPL34
TGGT1_212290	66	191.368	0	RPLS19
TGGT1_226970	67	190.18	0	RPS11
TGGT1_268200	68	189.006	83.321	RNA recognition motif-containing protein
TGGT1_237880	69	186.701	0	GRA13 (15)
TGGT1_224530	70	183.504	61.634	IMC5 (5)
TGGT1_202550	71	174.209	0	BCC6 (10)
TGGT1_291800	72	162.867	0	ADP-ribosylation factor family protein
TGGT1_242070	73	159.059	0	cAMP-dependent protein kinase regulatory subunit
TGGT1_273560	74	158.599	0	Putative kinesin heavy chain
TGGT1_219270	75	157.424	0	GAPM2a (16)
TGGT1_202930	76	155.689	0	ISC5 (7)
TGGT1_240630	77	153.095	0	IMC35 (this study)
TGGT1_229640	78	152.391	0	AC8 (3)
TGGT1_214880	79	147.997	0	AC4 (3)
TGGT1_224000	80	144.819	0	IAP5 (this study)
TGGT1_215470	81	141.101	0	RPL10A
TGGT1_269960	82	135.482	0	IMC40 (this study)
TGGT1_220930	83	133.707	0	ISC3 (3)
TGGT1_234380	84	129.376	0	MIC21 (17)
TGGT1_250340	85	127.049	0	Centrin 2 (18, 19)
TGGT1_248360	86	123.963	0	Hypothetical protein
TGGT1_294670	87	110.538	0	Translation initiation factor 3 subunit
TGGT1_206690	88	107.813	0	GAPM2b (16)
TGGT1_228290	89	107.813	0	Phospholipase/carboxylesterase
TGGT1_266060	90	106.686	0	Ribosomal protein RPSA
TGGT1_266090	91	101.837	0	Hypothetical protein
TGGT1_202500	92	100.39	0	GAPM1a (16)
TGGT1_232260	93	100.062	0	TSC5 (7)
TGGT1_226960	94	99.98	44.075	Phosphofructokinase PFKII
TGGT1_313010	95	96.691	0	DEAD (Asp-Glu-Ala-Asp) box polypeptide DDX6 (Ananvoranich, 2016)
TGGT1_248740	96	95.46	0	Localizes to the subpellicular microtubules
TGGT1_201760	97	91.128	0	TLAP4 (20)
TGGT1_297120	98	90.455	0	Hypothetical protein
TGGT1_226030	99	89.268	0	AGC kinase (PKAc1) (21)
TGGT1_285650	100	87.252	0	Hypothetical protein
TGGT1_311770	101	85.647	16.781	BCC3 (10 & this study)
TGGT1_310450	102	81.931	0	IAP2 (this study)
TGGT1_278850	103	81.433	0	DHHC2 (22)
TGGT1_221630	104	79.862	0	IAP4 (this study)
TGGT1_267370	105	75.977	0	Kinesin motor domain-containing protein (Kinesin A) (23)
TGGT1_212250	106	68.807	0	XPG N-terminal domain-containing protein
TGGT1_220900	107	65.054	0	AC13 (this study)
TGGT1_319850	108	62.744	0	Splicing factor U2AF family SnRNP auxilary factor large subunit
TGGT1_225690	109	62.234	0	AC7 (3)
TGGT1_240060	110	61.982	0	IST (24)
TGGT1_203010	111	60.873	0	Aurora Kinase (25)
TGGT1_222160	112	59.686	0	Aldehyde dehydrogenase
TGGT1_297870	113	55.004	0	IMC36 (this study)
TGGT1_225560	114	54.873	0	IMC41 (this study)
TGGT1_202220	115	53.343	0	Hypothetical protein
TGGT1_208030	116	52.791	0	MIC4 (26)
TGGT1_266630	117	52.61	0	CPH1 (14)
TGGT1_259940	118	49.148	0	Hypothetical protein
TGGT1_318440	119	47.971	0	Helicase associated domain (ha2) protein
TGGT1_232150	120	45.928	0	IMC32 (27)
TGGT1_249440	121	42.788	0	AC12 (this study)
TGGT1_312100	122	42.013	0	IMC42 (this study)
TGGT1_243960	123	38.418	0	Nuclear transport factor 2 (ntf2) domain-containing protein
TGGT1_315700	124	37.114	0	Hypothetical protein
TGGT1_310080	125	36.408	0	AMP-binding enzyme domain-containing protein
TGGT1_257430	126	34.021	0	Hypothetical protein
TGGT1_309600	127	31.895	0	Hypothetical protein
TGGT1_294860	128	31.155	0	BCC0 (10 & this study)
TGGT1_209050	129	31.117	0	TKL5 (28)
TGGT1_268870	130	29.698	0	Tetratricopeptide repeat-containing protein
TGGT1_255190	131	26.148	0	Myosin B and C (29)
TGGT1_307580	132	26.036	0	CBS domain-containing protein
TGGT1_243430	133	22.497	0	OTUD3C

TGGT1_298610	134	19.544	0	GYF domain-containing protein
TGGT1_225960	135	19.345	0	STE kinase
TGGT1_229260	136	17.699	0	BCC4 (10)
TGGT1_223920	137	15.775	0	RON3 (30)
TGGT1_231960	138	15.718	0	GRA28 (31)
TGGT1_278440	139	14.56	0	Nucleosome remodeling (32)

References for Table S2.

1. Mann T, Beckers C. 2001. Characterization of the subpellicular network, a filamentous membrane skeletal component in the parasite Toxoplasma gondii. Mol Biochem Parasitol 115:257–268.

2. Hu K, Johnson J, Florens L, Fraunholz M, Suravajjala S, DiLullo C, Yates J, Roos DS, Murray JM. 2006. Cytoskeletal Components of an Invasion Machine—The Apical Complex of Toxoplasma gondii. PLOS Pathogens 2:e13.

3. Chen AL, Kim EW, Toh JY, Vashisht AA, Rashoff AQ, Van C, Huang AS, Moon AS, Bell HN, Bentolila LA, Wohlschlegel JA, Bradley PJ. 2015. Novel Components of the Toxoplasma Inner Membrane Complex Revealed by BioID. mBio 6.

4. Lorestani A, Ivey FD, Thirugnanam S, Busby MA, Marth GT, Cheeseman IM, Gubbels M-J. 2012. Targeted proteomic dissection of Toxoplasma cytoskeleton sub-compartments using MORN1. Cytoskeleton (Hoboken) 69:1069–1085.

5. Anderson-White BR, Ivey FD, Cheng K, Szatanek T, Lorestani A, Beckers CJ, Ferguson DJP, Sahoo N, Gubbels M-J. 2011. A family of intermediate filament-like proteins is sequentially assembled into the cytoskeleton of Toxoplasma gondii. Cellular Microbiology 13:18–31.

6. Hu K, Johnson J, Florens L, Fraunholz M, Suravajjala S, DiLullo C, Yates J, Roos DS, Murray JM. 2006. Cytoskeletal Components of an Invasion Machine—The Apical Complex of Toxoplasma gondii. PLOS Pathogens 2:e13.

7. Chen AL, Moon AS, Bell HN, Huang AS, Vashisht AA, Toh JY, Lin AH, Nadipuram SM, Kim EW, Choi CP, Wohlschlegel JA, Bradley PJ. 2017. Novel insights into the composition and function of the Toxoplasma IMC sutures. Cellular Microbiology 19:e12678.

8. Beck JR, Rodriguez-Fernandez IA, de Leon JC, Huynh M-H, Carruthers VB, Morrissette NS, Bradley PJ. 2010. A novel family of Toxoplasma IMC proteins displays a hierarchical organization and functions in coordinating parasite division. PLoS Pathog 6:e1001094.

9. Dos Santos Pacheco N, Tosetti N, Krishnan A, Haase R, Maco B, Suarez C, Ren B, Soldati-Favre D. 2021. Revisiting the Role of Toxoplasma gondii ERK7 in the Maintenance and Stability of the Apical Complex. mBio 12:e02057-21.

10. Engelberg K, Bechtel T, Michaud C, Weerapana E, Gubbels M-J. 2022. Proteomic characterization of the Toxoplasma gondii cytokinesis machinery portrays an expanded hierarchy of its assembly and function. 1. Nat Commun 13:4644.

11. Tosetti N, Dos Santos Pacheco N, Bertiaux E, Maco B, Bournonville L, Hamel V, Guichard P, Soldati-Favre D. 2020. Essential function of the alveolin network in the subpellicular microtubules and conoid assembly in Toxoplasma gondii. eLife 9:e56635.

12. Frénal K, Marq J-B, Jacot D, Polonais V, Soldati-Favre D. 2014. Plasticity between MyoC- and MyoA-Glideosomes: An Example of Functional Compensation in Toxoplasma gondii Invasion. PLOS Pathogens 10:e1004504.

13. Beck JR, Fung C, Straub KW, Coppens I, Vashisht AA, Wohlschlegel JA, Bradley PJ. 2013. A Toxoplasma Palmitoyl Acyl Transferase and the Palmitoylated Armadillo Repeat Protein TgARO Govern Apical Rhoptry Tethering and Reveal a Critical Role for the Rhoptries in Host Cell Invasion but Not Egress. PLOS Pathogens 9:e1003162.

14. Long S, Anthony B, Drewry LL, Sibley LD. 2017. A conserved ankyrin repeat-containing protein regulates conoid stability, motility and cell invasion in Toxoplasma gondii. 1. Nature Communications 8:1–14.

15. Nadipuram SM, Kim EW, Vashisht AA, Lin AH, Bell HN, Coppens I, Wohlschlegel JA, Bradley PJ. 2016. In Vivo Biotinylation of the Toxoplasma Parasitophorous Vacuole Reveals Novel Dense Granule Proteins Important for Parasite Growth and Pathogenesis. mBio 7.

16. Harding CR, Egarter S, Gow M, Jiménez-Ruiz E, Ferguson DJP, Meissner M. 2016. Gliding Associated Proteins Play Essential Roles during the Formation of the Inner Membrane Complex of Toxoplasma gondii. PLOS Pathogens 12:e1005403.

17. Tagoe DNA, Drozda AA, Falco JA, Bechtel TJ, Weerapana E, Gubbels M-J. 2021. Ferlins and TgDOC2 in Toxoplasma Microneme, Rhoptry and Dense Granule Secretion. 3. Life 11:217.

18. Leung JM, Liu J, Wetzel LA, Hu K. 2019. Centrin2 from the human parasite Toxoplasma gondii is required for its invasion and intracellular replication. Journal of Cell Science 132:jcs228791.

19. Lentini G, Dubois DJ, Maco B, Soldati-Favre D, Frénal K. 2019. The roles of Centrin 2 and Dynein Light Chain 8a in apical secretory organelles discharge of Toxoplasma gondii. Traffic 20:583–600.

20. Liu J, He Y, Benmerzouga I, Sullivan WJ, Morrissette NS, Murray JM, Hu K. 2016. An ensemble of specifically targeted proteins stabilizes cortical microtubules in the human parasite Toxoplasma gondii. MBoC 27:549–571.

21. Sugi T, Ma YF, Tomita T, Murakoshi F, Eaton MS, Yakubu R, Han B, Tu V, Kato K, Kawazu S-I, Gupta N, Suvorova ES, White MW, Kim K, Weiss LM. 2016. Toxoplasma gondii Cyclic AMP-Dependent Protein Kinase Subunit 3 Is Involved in the Switch from Tachyzoite to Bradyzoite Development. mBio https://doi.org/10.1128/mBio.00755-16.

22. Frénal K, Tay CL, Mueller C, Bushell ES, Jia Y, Graindorge A, Billker O, Rayner JC, Soldati-Favre D. 2013. Global Analysis of Apicomplexan Protein S-Acyl Transferases Reveals an Enzyme Essential for Invasion. Traffic 14:895–911.

23. Leung JM, He Y, Zhang F, Hwang Y-C, Nagayasu E, Liu J, Murray JM, Hu K. 2017. Stability and function of a putative microtubule-organizing center in the human parasite Toxoplasma gondii. MBoC 28:1361–1378.

24. Olias P, Etheridge RD, Zhang Y, Holtzman MJ, Sibley LD. 2016. Toxoplasma Effector Recruits the Mi-2/NuRD Complex to Repress STAT1 Transcription and Block IFN-γ-Dependent Gene Expression. Cell Host & Microbe 20:72–82.

25. Berry L, Chen C-T, Reininger L, Carvalho TG, El Hajj H, Morlon-Guyot J, Bordat Y, Lebrun M, Gubbels M-J, Doerig C, Daher W. 2016. The conserved apicomplexan Aurora kinase TgArk3 is involved in endodyogeny, duplication rate and parasite virulence. Cellular Microbiology 18:1106–1120.

26. Brecht S, Carruthers VB, Ferguson DJP, Giddings OK, Wang G, Jäkle U, Harper JM, Sibley LD, Soldati D. 2001. The Toxoplasma Micronemal Protein MIC4 Is an Adhesin Composed of Six Conserved Apple Domains *. Journal of Biological Chemistry 276:4119–4127.

27. Torres JA, Pasquarelli RR, Back PS, Moon AS, Bradley PJ. 2021. Identification and Molecular Dissection of IMC32, a Conserved Toxoplasma Inner Membrane Complex Protein That Is Essential for Parasite Replication. mBio 12.

28. Varberg JM, Coppens I, Arrizabalaga G, Gaji RY. 2018. TgTKL1 Is a Unique Plant-Like Nuclear Kinase That Plays an Essential Role in Acute Toxoplasmosis. mBio 9:e00301-18.

29. Delbac F, Sänger A, Neuhaus EM, Stratmann R, Ajioka JW, Toursel C, Herm-Götz A, Tomavo S, Soldati T, Soldati D. 2001. Toxoplasma gondii myosins B/C : one gene, two tails, two localizations, and a role in parasite division. Journal of Cell Biology 155:613–624.

30. Bradley PJ, Ward C, Cheng SJ, Alexander DL, Coller S, Coombs GH, Dunn JD, Ferguson DJ, Sanderson SJ, Wastling JM, Boothroyd JC. 2005. Proteomic Analysis of Rhoptry Organelles Reveals Many Novel Constituents for Host-Parasite Interactions in Toxoplasma gondii*. Journal of Biological Chemistry 280:34245–34258.

31. Rudzki EN, Ander SE, Coombs RS, Alrubaye HS, Cabo LF, Blank ML, Gutiérrez-Melo N, Dubey JP, Coyne CB, Boyle JP. 2021. Toxoplasma gondii GRA28 Is Required for Placenta-Specific Induction of the Regulatory Chemokine CCL22 in Human and Mouse. mBio 12:e01591-21.

32. Syn G, Blackwell JM, Jamieson SE, Francis RW. 2018. An in silico pipeline to filter the Toxoplasma gondii proteome for proteins that could traffic to the host cell nucleus and influence host cell epigenetic regulation. Mem Inst Oswaldo Cruz 113.
