# Supplementary material for: IMC29 Plays an Important Role in Toxoplasma Endodyogeny and Reveals New Components of the Daughter-Enriched IMC Proteome
Source: mBio. 2023 Jan 9;14(1):e03042-22. doi: 10.1128/mbio.03042-22 (PMC9973257; doi:10.1128/mbio.03042-22)
Supplement: TABLE S3 [file mbio.03042-22-s0010.pdf]

**Table S3: Oligonucleotides used in this study.**

| Purpose | Name | Description | Sequence 5'-3' |
|---|---|---|---|
| **IMC29 (TGGT1_243200) tagging** | P1 | IMC29 gRNA-tagging sense | AAGTTGCTCCGTTTCTGGTGTGTCTG |
| | P2 | IMC29 gRNA-tagging antisense | AAAACAGACACACCAGAAACGGAGCA |
| | P3 | IMC29 HDR tagging template fwd | CTCACGCTCAGGCAGCAGTACCCCGGACACGGCCTCAATGGAAGTGGAGGACGGGAATTC |
| | P4 | IMC29 HDR tagging template rev | GTCACATCCGTATACACGGTTCTATGAGCAGATTCCGTCGACGGCCAGTGAATTGTAATA |
| **IMC29 (TGGT1_243200) knockout** | P5 | IMC29 gRNA-KO sense | AAGTTgTAAGGGTGCAAGAGTTGATGG |
| | P6 | IMC29 gRNA-KO antisense | AAAACCATCAACTCTTGCACCCTTAcA |
| | P7 | IMC29 HDR KO template fwd | GCTGAAGACTCGCCTGATAAAGCTCACGAAGGCAAACACGGGAAGTGGAGGACGGGAATT |
| | P8 | IMC29 HDR KO template rev | AGTCCACTGCTGCTCCTCGAGCAGTCTGGGAGATTTGGCGACGGCCAGTGAATTGTAATA |
| **IMC29 (TGGT1_243200) knockout regenerated** | P9 | IMC29 gRNA-KO sense new | AAGTTGAACGATGAGCCGAGCAAGGG |
| | P10 | IMC29 gRNA-KO antisense new | AAAACCCTTGCTCGGCTCATCGTTCA |
| | P11 | IMC29 HDR KO template fwd new | GCCCAAGCAGAGCTGCTGCCGTTTGGTGGGTCAGGAATCCCACTCCATGGAACCTGACTG |
| | P12 | IMC29 HDR KO template rev new | GATTCCTGACCCACCAAACGGCAGCAGCTCTGCTTGGGCCCTGCAAGTGCATAGAAGGAA |
| **AC9 (TGGT1_246950) tagging** | P13 | AC9 gRNA-tagging sense | AAGTTATGTGTTCCTCAAATGTCAGG |
| | P14 | AC9 gRNA-tagging antisense | AAAACCTGACATTTGAGGAACACATA |
| | P15 | AC9 HDR template fwd | GTCGGGGAACCGCGAACCGAGTGAATATCCGCAGGGAATGGAAGTGGAGGACGGGAATTC |
| | P16 | AC9 HDR template rev | CTCGCAGCGTGTCGCACTGAACTCTTGTGCGGAGAGAGCGACGGCCAGTGAATTGTAATA |
| **FBXO1 (TGGT1_310930) tagging** | P17 | FBXO1 gRNA-tagging sense | AAGTTAGCCTTCTGGAGCACGGCAGG |
| | P18 | FBXO1 gRNA-tagging antisense | AAAACCTGCCGTGCTCCAGAAGGCTA |
| | P19 | FBXO1 HDR template fwd | CATAATCGATTACTACATGAGTCAGGGCGGAGCCAATGCGGGAAGTGGAGGACGGGAATT |
| | P20 | FBXO1 HDR template rev | GGTATCAGATCATCGAGAAACAAACGTTCGATGCTGCTCCCGACGGCCAGTGAATTGTAATA |
| **IMC32 (TGGT1_232150) tagging** | P21 | IMC32 gRNA-tagging sense | AAGTTATGCTGTGGCGGAGGAAGTTG |
| | P22 | IMC32 gRNA-tagging antisense | AAAACAACTTCCTCCGCCACAGCATA |
| | P23 | IMC32 HDR template fwd | GCTGAAGACTCGCCTGATAAAGCTCACGAAGGCAAACACGGGAAGTGGAGGACGGGAATT |
| | P24 | IMC32 HDR template rev | AGTCCACTGCTGCTCCTCGAGCAGTCTGGGAGATTTGGCGACGGCCAGTGAATTGTAATA |
| **IMC29 full length cDNA** | P25 | IMC29 full length fwd (BglII) | AGATCTATGGAAGCGTTGCCTGTCAAC |
| | P26 | IMC29 full length rev (NotI) | gcggccgcATTGAGGCCGTGTCCGGG |
| **IMC29 endogenous promoter** | P27 | IMC29 promoter fwd (NsiI) | ATGCATCACTACCACACCGTTTTATCAACT |
| | P28 | IMC29 promoter rev (BglII) | AGATCTTTTCGACAGGTCTTTCTGTTTAAAGT |
| **IMC29 deletion construct (Δ2-100)** | P29 | IMC29 2-100 fwd (BglII) | AGATCTATGGGCGAGCGGGAAGGTCTTTC |
| **IMC29 deletion construct (Δ2-137)** | P30 | IMC29 2-137 fwd (BglII) | AGATCTATGGCTCTGGCCAAAGCGGCAG |
| **IMC29 deletion construct (Δ2-199)** | P31 | IMC29 2-199 fwd (BglII) | AGATCTatgGCCCCAAGCAAGCAGAGCTC |
| **IMC29 deletion construct (Δ2-389)** | P32 | IMC29 2-389 fwd (BglII) | AGATCTATGCTCCTCACGCTTGCGAAGAAAGC |
| **IMC29 deletion construct (Δ2-500)** | P33 | IMC29 2-500 fwd (BglII) | AGATCTATGGGTATTCCTTATTCCACCGTTCT |
| **IMC29 deletion construct (Δ1111-1258)** | P34 | IMC29 1111-1258 rev (NotI) | gcggccgcGACATCCTGGAAGGACGACA |
| **IMC29 deletion construct (Δ1218-1258)** | P35 | IMC29 1218-1258 rev (NotI) | gcggccgcGCTCCGTACAGCCGCTTGA |
| **IMC29 deletion construct (Δ1111-1218)** | P36 | IMC29 1111-1218 fwd (Q5) | GAAACGCCAGGAAGAGCA |
| | P37 | IMC29 1111-1218 rev (Q5) | GACATCCTGGAAGGACGAC |
| **TgGT1_294610 epitope tagging** | P38 | 294610 gRNA-tagging sense | AAGTTGGAGAGTTTCGACAGTGCTTG |
| | P39 | 294610 gRNA-tagging antisense | AAAACAAGCACTGTCGAAACTCTCCA |
| | P40 | 294610 HDR template fwd | GCACTCTCAATACAAGATGGTCTTCCGACAGGGTCTCCCAGGAAGTGGAGGACGGGAATT |
| | P41 | 294610 HDR template rev | TACCTGTTGAGAAATCCCTTCATCATAGGAAGATAGTGCGACGGCCAGTGAATTGTAATA |
| **TgGT1_255450 epitope tagging** | P42 | 255450 gRNA-tagging sense | AAGTTgCTTGTCCACCTCTCAAACTCG |
| | P43 | 255450 gRNA-tagging antisense | AAAACGAGTTTGAGAGGTGGACAAGcA |
| | P44 | 255450 HDR template fwd | CTTCTGGACGCAAATGTGCAAGCACCTAGAGACTGCTCAGGGAAGTGGAGGACGGGAATT |
| | P45 | 255450 HDR template rev | GGTTTAACGAATGTGAGTTTCAGATGACTCGCAGAAGTCGACGGCCAGTGAATTGTAATA |
| **TgGT1_240630 epitope tagging** | P46 | 240630 gRNA-tagging sense | AAGTTATGGCGTGGCAGGATAGCCGG |
| | P47 | 240630 gRNA-tagging antisense | AAAACCGGCTATCCTGCCACGCCATA |
| | P48 | 240630 HDR template fwd | TGCACCGCCGAAGCCAAGAGGTTACCAGTATTAAGATGTGGAAGTGGAGGACGGGAATTC |
| | P49 | 240630 HDR template rev | AATGTCTTGTCTGCCGACCGCGAAACGACGCATCAGACCGACGGCCAGTGAATTGTAATA |
| **TgGT1_294860 epitope tagging** | P50 | 294860 gRNA-tagging sense | AAGTTGCAGTGCTAGCGGGGAGACGG |
| | P51 | 294860 gRNA-tagging antisense | AAAACCGTCTCCCCGCTAGCACTGCA |
| | P52 | 294860 HDR template fwd | GGGGGGCGCAGGGAAGCTGGGGAGACAAGTGACGTTTTACGGAAGTGGAGGACGGGAATT |
| | P53 | 294860 HDR template rev | CGCCGATACAAATGACGGACCCGAGACAGACGCGAGAACGACGGCCAGTGAATTGTAATA |
| **TgGT1_311770 epitope tagging** | P54 | 311770 gRNA-tagging sense | AAGTTGTTGTCTCAGTCCACTCTTGG |
| | P55 | 311770 gRNA-tagging antisense | AAAACCAAGAGTGGACTGAGACAACA |
| | P56 | 311770 HDR template fwd | GGGCAGATGGAAAGACAGGTATAAATCCGCAAGAGGCCTCGGAAGTGGAGGACGGGAATT |
| | P57 | 311770 HDR template rev | CTCCCCAGAGAAATTTTCTACATACTCCTGTACAGACCCGACGGCCAGTGAATTGTAATA |
| **TgGT1_297870 epitope tagging** | P58 | 297870 gRNA-tagging sense | TTCCGTGGCGACGACACAAGAAGACGTTACTTCCGTCAACGGAAGTGGAGGACGGGAATT |
| | P59 | 297870 gRNA-tagging antisense | TACAAAGAAAATCACAATTCGACTGACTCAGCTTCCTGCGACGGCCAGTGAATTGTAATA |
| | P60 | 297870 HDR template fwd | AAGTTgCAACAAGAGCCACTGCACGGG |
| | P61 | 297870 HDR template rev | AAAACCCGTGCAGTGGCTCTTGTTGcA |
| **TgGT1_249440 epitope tagging** | P62 | 249440 gRNA-tagging sense | AAGTTGCAGTGGAACATGAGTCGCAG |
| | P63 | 249440 gRNA-tagging antisense | AAAACTGCGACTCATGTTCCACTGCA |
| | P64 | 249440 HDR template fwd | TGGTGGAGGAATTTCCGCCGCTGAACCTCAATTTTTACTCGGAAGTGGAGGACGGGAATT |
| | P65 | 249440 HDR template rev | CAGACGGACTTGGGTAAAGCACCACTACACAACTGCTCCGACGGCCAGTGAATTGTAATA |
| **TgGT1_220900 epitope tagging** | P66 | 220900 gRNA-tagging sense | AAGTTGGGTAGATCTGATGCTTCCAG |
| | P67 | 220900 gRNA-tagging antisense | AAAACTGGAAGCATCAGATCTACCCA |
| | P68 | 220900 HDR template fwd | TGCGCCCACAGAGTACTCCCTTGAAATTGAGAAAGCATTCGGAAGTGGAGGACGGGAATT |
| | P69 | 220900 HDR template rev | CCTGAGGTGTACAGCACAGGCGGCCACTGCTCAGACACCGACGGCCAGTGAATTGTAATA |
| **TgGT1_310450 epitope tagging** | P70 | 310450 gRNA-tagging sense | AAGTTgTCTGTGAACATGTCAGTTCGG |
| | P71 | 310450 gRNA-tagging antisense | AAAACCGAACTGACATGTTCACAGAcA |
| | P72 | 310450 HDR template fwd | GCGCTCGGAGTTTCATCCTGTCACACCAGGTGTTCGGTGGGGAAGTGGAGGACGGGAATT |
| | P73 | 310450 HDR template rev | GAAGATTGTGCATTTCCATATCTCCAGCAAAGACTCTGCGACGGCCAGTGAATTGTAATA |
| | P74 | 238170 gRNA-tagging sense | AAGTTATATGATGACGAAGGGATGTG |

| | | | |
|---|---|---|---|
| **TgGT1_238170 epitope tagging** | P75 | 238170 gRNA-tagging antisense | AAAACACATCCCTTCGTCATCATATA |
| | P76 | 238170 HDR template fwd | CACGAAATCGCGTCTCTTCTCGCTCTTACACGACCTTCTTGGAAGTGGAGGACGGGAATT |
| | P77 | 238170 HDR template rev | ATACAAATCGACAAGAAATCACAAATCGAGACAGATCCCGACGGCCAGTGAATTGTAATA |
| **TgGT1_221630 epitope tagging** | P78 | 221630 gRNA-tagging sense | AAGTTACAACGTTCTCACTTCGTTCG |
| | P79 | 221630 gRNA-tagging antisense | AAAACGAACGAAGTGAGAACGTTGTA |
| | P80 | 221630 HDR template fwd | TGGACCCCAGCAATCGTGCTGTGACAGTGACGGATCTTCTGGAAGTGGAGGACGGGAATT |
| | P81 | 221630 HDR template rev | CCGTACGAGACAACACCACAACGACCCTCGAAGCAGTCCGACGGCCAGTGAATTGTAATA |
| **TgGT1_224000 epitope tagging** | P82 | 224000 gRNA-tagging sense | AAGTTGAGCAACAAGATGCGCAAACG |
| | P83 | 224000 gRNA-tagging antisense | AAAACGTTTGCGCATCTTGTTGCTCA |
| | P84 | 224000 HDR template fwd | AACTGGCGGCGAGGGGCCTGACATAAAAGGACTCGTCCTTGGAAGTGGAGGACGGGAATT |
| | P85 | 224000 HDR template rev | TACTCCTGAGTGAATCAAGAAAACCTTTCACTTCTGCCCGACGGCCAGTGAATTGTAATA |
| **TgGT1_235690 epitope tagging** | P86 | 235690 gRNA-tagging sense | AAGTTGTAAGACTACCGTTGCGGTAG |
| | P87 | 235690 gRNA-tagging antisense | AAAACTACCGCAACGGTAGTCTTACA |
| | P88 | 235690 HDR template fwd | ACACAAGGCTCGCTCTCATAAGAGGAAATTGATTTGTTGCGGAAGTGGAGGACGGGAATT |
| | P89 | 235690 HDR template rev | ACTCTAGAGCGTGGCAGAAATGTGTCCTTTCTCTGGACCGACGGCCAGTGAATTGTAATA |
| **TgGT1_293360 epitope tagging** | P90 | 293360 gRNA-tagging sense | AAGTTGGACTTCTAGGGATTGTTGGG |
| | P91 | 293360 gRNA-tagging antisense | AAAACCCAACAATCCCTAGAAGTCCA |
| | P92 | 293360 HDR template fwd | GTTTCGCAGCATGCTCCGGCCGTCACAGATCGATCTATTCGGAAGTGGAGGACGGGAATT |
| | P93 | 293360 HDR template rev | TGACATGGACAAAACGCTGACATTCCGTGAGGCAATTCCGACGGCCAGTGAATTGTAATA |
| **TgGT1_255420 epitope tagging** | P94 | 255420 gRNA-tagging sense | AAGTTGGTTCCTCTATCCAGTGACGG |
| | P95 | 255420 gRNA-tagging antisense | AAAACCGTCACTGGATAGAGGAACCA |
| | P96 | 255420 HDR template fwd | AGGAGAAGGGGCTGCGGAATTGCTGGAGTCTCTTAATGCAGGAAGTGGAGGACGGGAATT |
| | P97 | 255420 HDR template rev | AAGAAGATTCTGCTGCGAACGTGTGCGGTCGATCTTCCCGACGGCCAGTGAATTGTAATA |
| **TgGT1_269960 epitope tagging** | P98 | 269960 gRNA-tagging sense | AAGTTgATCCCCGTTTCTGCGCTTCGG |
| | P99 | 269960 gRNA-tagging antisense | AAAACCGAAGCGCAGAAACGGGGATcA |
| | P100 | 269960 HDR template fwd | TGGACAATCTCTCGACGCAGGGAGCACCGCTTCTTTCGCCGGAAGTGGAGGACGGGAATT |
| | P101 | 269960 HDR template rev | TCCGCCTCTCTGCTCTTTGCCCGGTGGCGCGTTCCTTTCGACGGCCAGTGAATTGTAATA |
| **TgGT1_225560 epitope tagging** | P102 | 225560 gRNA-tagging sense | AAGTTGAAGTGTCTTTCGACGAATAG |
| | P103 | 225560 gRNA-tagging antisense | AAAACTATTCGTCGAAAGACACTTCA |
| | P104 | 225560 HDR template fwd | GTCCACTCGTGAGGAGAACATAAACGACTTTTTTCACGATGGAAGTGGAGGACGGGAATT |
| | P105 | 225560 HDR template rev | GACACCACATTTCACAAAGTGGCATCGAATGATCAGGGCGACGGCCAGTGAATTGTAATA |
| **TgGT1_312100 epitope tagging** | P106 | 312100 gRNA-tagging sense | AAGTTGCACACGAAAGAAACGTCTGG |
| | P107 | 312100 gRNA-tagging antisense | AAAACCAGACGTTTCTTTCGTGTGCA |
| | P108 | 312100 HDR template fwd | ACTCAGTGACGCCAGGGAGGAGGCATGCATAGAAATGCAAGGAAGTGGAGGACGGGAATT |
| | P109 | 312100 HDR template rev | GCGCCCCTGTTCTCAAAACTCTTCCGTTCCCCTTGTCGCGACGGCCAGTGAATTGTAATA |
| **TgGT1_311230 epitope tagging** | P110 | 311230 gRNA-tagging sense | AAGTTgTTTGTAGTGGCGGCGAACATG |
| | P111 | 311230 gRNA-tagging antisense | AAAACATGTTCGCCGCCACTACAAAcA |
| | P112 | 311230 HDR template fwd | GCAGCCTCGTGGTTCCCGCCGGAAGAAGAGGGCTGCGAGGGAAGTGGAGGACGGGAATTC |
| | P113 | 311230 HDR template rev | CACGGTTTATCTCGAAGTTGACGATGGGGCAGAACCACCGACGGCCAGTGAATTGTAATA |
| **TgGT1_202550 epitope tagging** | P114 | 202550 gRNA-tagging sense | AAGTTgAAAGTCGCTCGCGACCCATGG |
| | P115 | 202550 gRNA-tagging antisense | AAAACCATGGGTCGCGAGCGACTTTcA |
| | P116 | 202550 HDR template fwd | GCGGTGGGGAGGAAGTAAAGGCAAGGACGCACAAAGGCGCGGAAGTGGAGGACGGGAATT |
| | P117 | 202550 HDR template rev | TCCGAGACATGGGACTCATCGTCTCCAAAAACAACGCGCGACGGCCAGTGAATTGTAATA |
| **TgGT1_229260 epitope tagging** | P118 | 229260 gRNA-tagging sense | AAGTTGCGCGGCCAACGGACGCTGTG |
| | P119 | 229260 gRNA-tagging antisense | AAAACACAGCGTCCGTTGGCCGCGCA |
| | P120 | 229260 HDR template fwd | CTACAGAAACGGCGTTCCTGCCTCTCTTGCAAATGAAATCGGAAGTGGAGGACGGGAATT |
| | P121 | 229260 HDR template rev | TCTACAGAAGAGAGACCCTTGGTCTGTCTATACACCAGCGACGGCCAGTGAATTGTAATA |
